# Contrastive learning unites sequence and structure in a global representation of protein space

**DOI:** 10.1073/pnas.2532702123

**Published:** 2026-08-03

**Authors:** Guy Yanai, Gabriel Axel, Liam M. Longo, Nir Ben-Tal, Rachel Kolodny

**Affiliations:** ^a^ Department of Computer Science, University of Haifa, Haifa 3303221, Israel; ^b^ Department of Biochemistry and Molecular Biology, School of Neurobiology, Biochemistry and Biophysics, George S. Wise Faculty of Life Sciences, Tel Aviv University, Ramat Aviv 6997801, Israel; ^c^ Earth-Life Science Institute, Institute of Science Tokyo, Tokyo 152-8550, Japan; ^d^ Blue Marble Space Institute of Science, Seattle, WA 98104

**Keywords:** contrastive learning, protein sequence and structure, protein domains, protein universe, maps of protein space

## Abstract

Establishing a coherent mapping of the relationships among all known proteins is crucial for elucidating processes of protein emergence and evolution. Yet the capacity to fully capture relationships of protein similarity is complicated by the nonstraightforward interplay between sequence and structure; indeed, proteins with unrelated sequences can adopt similar structures, and, conversely, proteins with similar or identical sequences can manifest radically different structures. Here, we introduce Contrastive Learning Sequence–Structure (CLSS), a contrastive protein language model (PLM) trained to coembed sequence and structure information in a self-supervised manner, facilitating a holistic representation of protein relatedness. CLSS represents the structures and sequences of full domains and domain subsequences as vectors in the same high-dimensional latent space. We show that this approach yields meaningful shared representations, which recapitulate the extensive structure- and sequence-based knowledge encoded in human-curated hierarchical protein classification systems (ECOD and CATH). Moreover, the representations generated by CLSS outperform those generated by alternative state-of-the-art PLMs in downstream classification tasks. Notably, we show that even the far larger space of domain subsequences is successfully coembedded, establishing a PLM tailored to these evolutionarily meaningful objects. CLSS embeddings produce informative representations of the protein universe without further downstream processing, as we demonstrate by analyzing preferential associations between protein architectures and ligand types across protein space.

## Significance

How can the relationships between all known proteins be represented? Recent advances in AI describe proteins as vectors, where the distance between vectors relates to the similarity between proteins. These vector representations are largely based on data regarding protein sequences or structures alone, neglecting the complex sequence–structure interplay. We introduce an AI model that generates representations integrating both sequence- and structure-based knowledge; it also incorporates information on subsequences, which are evolutionarily meaningful. We use the model to generate holistic maps of protein similarity, revealing that such joint mapping is possible and that it captures known (expert-curated) relationships between proteins. Moreover, our joint sequence–structure-based representations of proteins are more accurate than other AI-based representations, as reflected in performance in downstream tasks.

Mapping the protein universe—that is, constructing representations of the relationships between all proteins—is essential to understanding the forces that have shaped protein emergence and evolution over the past 4 billion years (1–3). To date, the most significant mapping achievements are hierarchical classifications (4–7), where building blocks of protein tertiary structure, called domains, are grouped based on sequence and/or structure similarity. Hierarchical classification databases are constructed under expert supervision, meaning that humans determine criteria for grouping specific domains together. Accordingly, these databases encompass a wealth of human insight and intuition regarding protein relatedness and similarity. At the same time, however, they accommodate only restricted definitions of these concepts. Specifically, hierarchical classifications coarse-grain interdomain relationships into just a few nested tiers and prioritize full-domain (global) evolutionary relationships, effectively neglecting similarities that are confined to smaller, subdomain-sized protein segments. These limitations can obscure evolutionary information, given that similar sequence segments that are present in different domains (8), including segments that bridge different folds (9–11), can hint at early evolutionary events (12, 13). These shared segments highlight fragment repurposing and recontextualization in the functional evolution of folds.

Alternative mapping approaches that capture more subtle interdomain relationships include networks (3, 14–16) and Euclidean maps (17–30). Specifically, a network is constructed from pairwise comparisons across domains, where two domains are connected if they share a subdomain-sized segment exceeding a predetermined length. This approach can reveal cross-fold similarities (and, thus, potential early evolutionary relationships) even in domains from ostensibly different evolutionary lineages (8–11, 31). Euclidean maps, on the other hand, can now be generated by Protein Language Models (PLMs) (25, 26, 28, 30, 32–34)—state-of-the-art computational models that are trained by self-supervision from vast amounts of data.

Whereas networks and Euclidean maps primarily describe either sequence- or structure-based similarity, respectively, we suggest that a meaningful overview of the protein universe must provide a unified account of both forms of similarity, while accommodating the many-to-many relationship between them (35–37). Sequence and structure carry complementary information that captures different evolutionary timescales and processes. The inclusion of both modalities is crucial to understanding both local and global evolutionary relationships. Indeed, it is for this reason that expert curation integrates both sequence and structure similarity (4–7).

We hypothesize that coembedding sequence and structure modalities with state-of-the-art deep learning methods will allow paired data to guide one another, improving protein mapping tasks. Contrastive Language-Image Pretraining (CLIP) (38), for example, coembeds images and their natural language text descriptions. In CLIP, contrastive loss (CL) guides the image-text coembedding, enabling state-of-the-art classification of images based on textual descriptions. We therefore propose that the application of CL, and its pairing with PLMs (23, 24), presents a unique opportunity to explicitly consider the complex interplay between sequence and structure. Moreover, given the potential of subdomain-sized sequence fragments to provide clues into distant evolutionary relationships between folds, we seek to explore the implications of directly incorporating subsequence representations into the CL training procedure.

Here, we report Contrastive Learning Sequence–Structure (CLSS), a PLM that embeds domain sequences or sequence fragments alongside domain structures. To test whether coembedding is possible and improves the representation of protein space in comparison with current approaches, we compare CLSS embeddings to those of three state-of-the-art PLMs that can embed both sequences and structures: ESM3 (25) and ProstT5 (39), which were trained to embed either sequences or structures individually; and ProTrek (30), which was trained to coembed both. We note that, when generating an embedding, each trained model, including CLSS, receives as input a single modality (i.e., a sequence, sequence fragment, or structure). We demonstrate that CLSS is the only PLM that successfully coembeds the three modalities in a cohesive map, thereby jointly representing structure- and sequence-based similarity. To further compare the PLMs’ performance, we apply them to downstream classification tasks from ProteinShake (40), a standardized package designed for evaluating deep learning protein models. We find that CLSS is a top performer and that it outperforms both of its parent models (the ESM3 structure encoder and the ESM2 sequence encoder), demonstrating the value of coembedding. Encouragingly, CLSS yields excellent agreement with curated labels across all levels of hierarchy in two prominent hierarchical classification databases: ECOD and CATH. Crucially, curated labels from these databases were not used during training. We conclude that maps constructed from CLSS embeddings can provide a holistic, structure- and sequence-aware view of the protein universe.

## Results

### The CLSS Architecture.

**Fig. 1.**
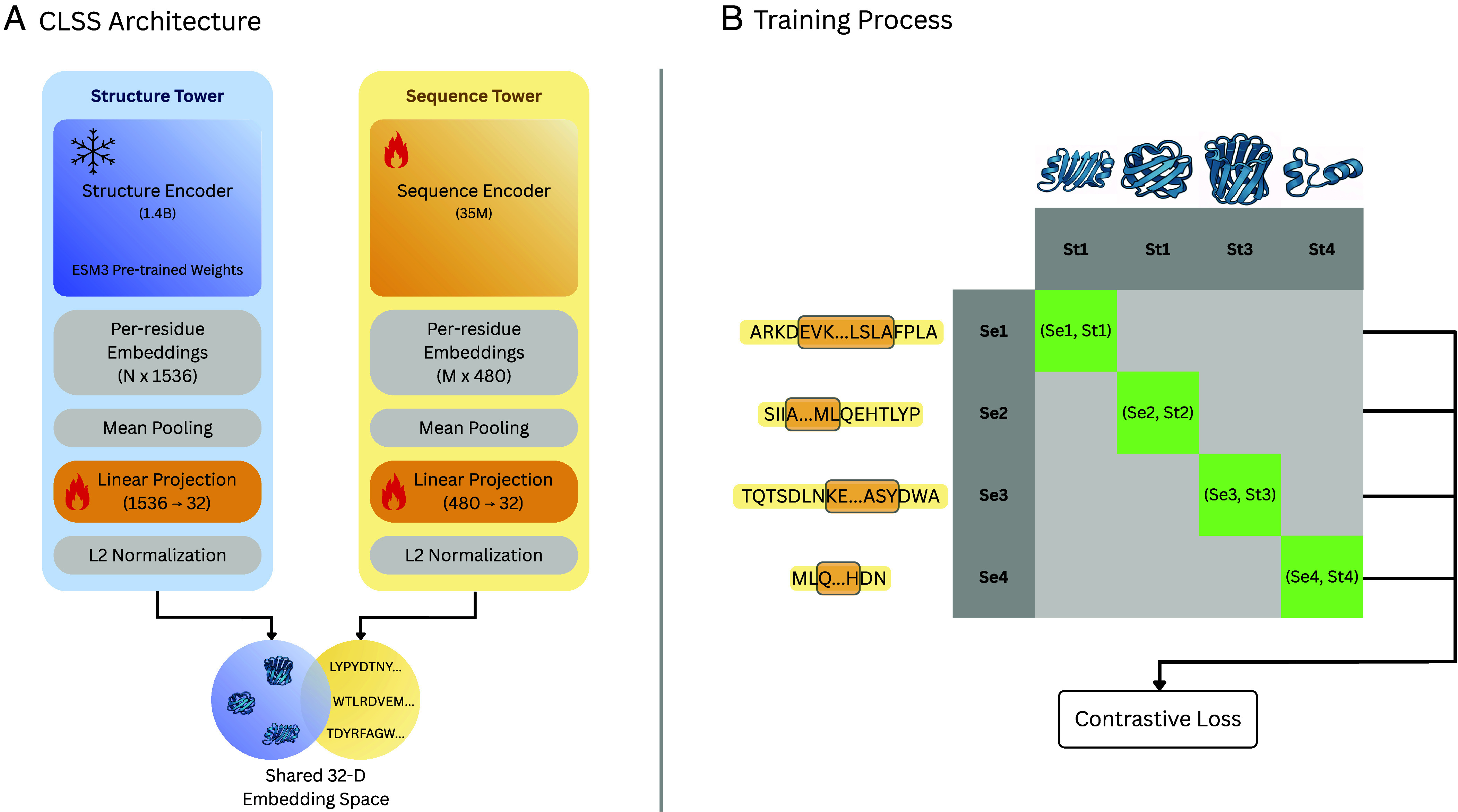
The CLSS architecture and training process. (*A*) CLSS has two towers: the sequence tower (ESM2-like architecture and initial weights) and its adapter network, and the structure tower (frozen ESM3 architecture and weights) and its adapter network. (*B*) During training, we calculated the embeddings using both towers. For CLSS-sub, a sampled subsequence (marked by boxes) was paired with the full domain structure. We then optimized the contrastive loss, which aims to maximize the dot-product similarities between embeddings corresponding to the same domain (marked in green), while minimizing other dot-product similarities (gray), using cross-entropy.

We designed and trained CLSS with two motivations: 1) to coembed domain sequences alongside domain structures, and 2) to meaningfully coembed subsequences (Fig. 1). CLSS is a deep network with two towers (38, 41), one for structure and one for sequence. The structure tower is based on the frozen (untrained) ESM3 structure encoder (25), which was trained to reproduce masked amino acids, structure tokens, and several other modalities in a given protein using the Masked Language Modeling (MLM) objective. The sequence tower has the same architecture and initial weights as the ESM2 35-million-parameter model, originally trained with a similar MLM objective, only confined to protein sequences. Both encoders are followed by a trained adapter that averages the embeddings, reduces their size, and normalizes the output. Models were trained for 80 epochs, during which contrastive loss was minimized for batches of 180 domains. The training dataset (1 million domains, including both sequences and structures; see *Methods*) was randomly sampled from the ECOD database, which includes both experimentally determined structures and AlphaFold2 (AF) predictions (42). Training was entirely self-supervised, using only the sequences and structures of the domains.

Two key models were trained: CLSS-full, in which full domain structures were contrasted against full domain sequences; and CLSS-sub, in which full domain structures were contrasted against contiguous subsequences. The embedding dimension, temperature, and minimal subsequence length were varied during the hyperparameter search. To motivate the final hyperparameter selection, we measured the self-consistency of the coembedding on a validation set of 50,000 domains (*SI Appendix*, Figs. S1–S3; see *Methods* for more details). Note that the self-consistency of embeddings achieved by CLSS-full is higher than that of CLSS-sub. Only CLSS-sub, however, was trained to represent subsequences. It is important to note that the trained model receives as input either a single sequence (or sequence fragment) or structure, on the basis of which it calculates an embedding; however, the resulting calculated embedding reflects knowledge learned from the self-supervised pairing of sequence and structure in the training phase.

### CLSS Unifies Sequence Space and Structure Space.

**Fig. 2.**
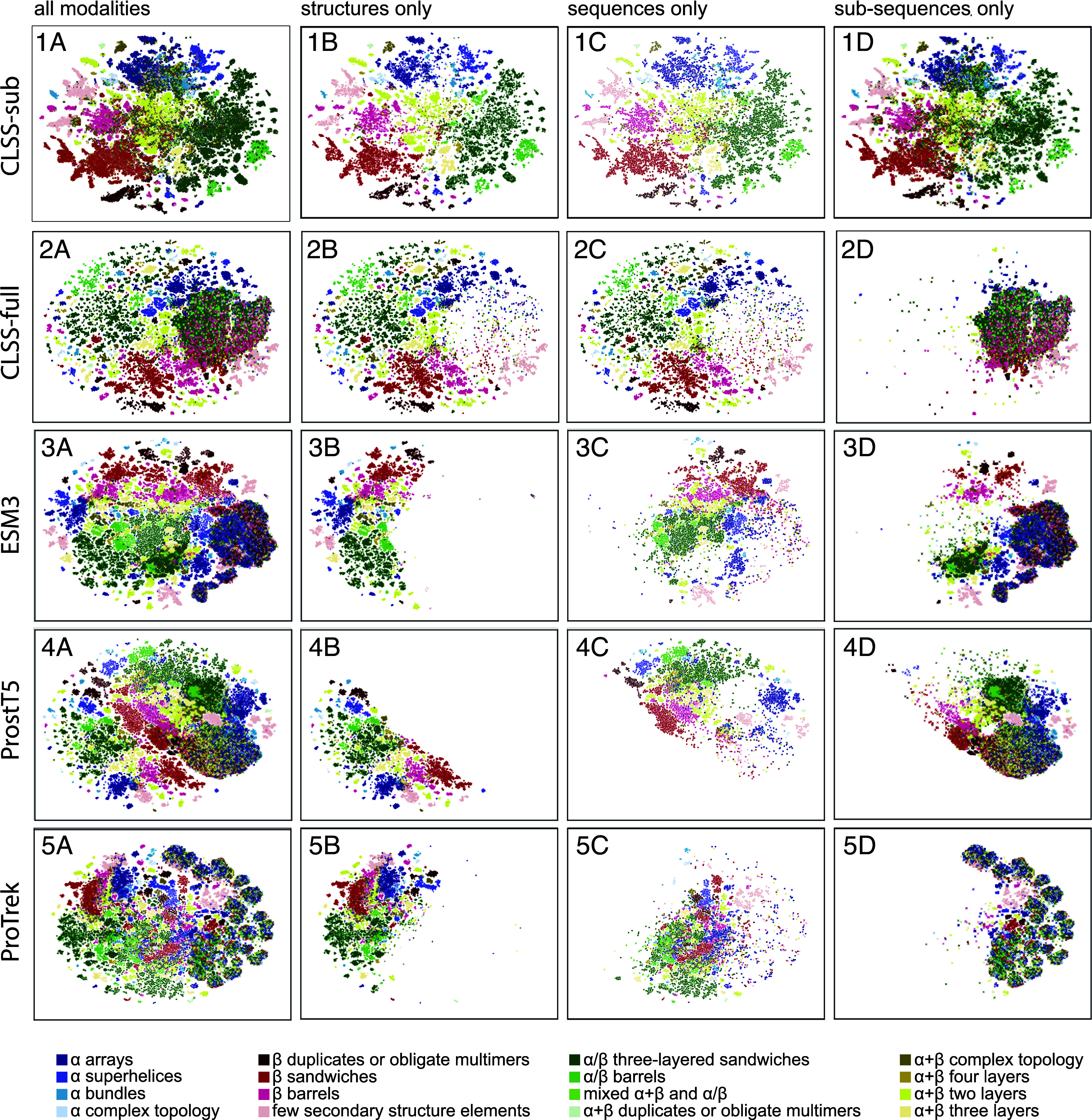
Comparison of maps generated for embeddings of ECOD domains (Dataset S1) with CLSS-sub, CLSS-full, ESM3, ProstT5, and ProTrek (arranged *Top* to *Bottom*). For each model, we calculated the embeddings with each of three input modalities—structure, sequence, and subsequence—and computed the t-SNE projection of the dataset of all embeddings simultaneously. Each point represents an embedding generated for one of the modalities of a domain, colored according to its ECOD architecture. (*A*) Map of all three modalities. For clarity we also show (*B*) structure embeddings, (*C*) sequence embeddings, and (*D*) subsequence embeddings alone. *SI Appendix*, Figs. S3–S7 show enlarged views of the embeddings of each model. We find that in ESM3, ProstT5, and ProTrek, embeddings of different modalities occupy distinct regions of the jointly projected map. In CLSS-full, the full sequence and structure modalities significantly overlap, but the subsequence modality occupies a separate region of the space. In CLSS-sub, all three modalities overlap and form similar maps.

Using the trained models, we calculated sequence- and structure-based embeddings for three sets of representative domains taken from across the protein universe: Dataset S1 comprises 31,696 ECOD domains from the 109 most-populated protein evolutionary lineages (ECOD X-group), spanning 16 architectures; Dataset S2 comprises 34,653 CATH (S40) domains; and Dataset S3 comprises 9,899 CATH domains from Røgen’s set of sequence-similar but topologically distinct domain pairs (36). For each domain, using each model, we separately calculated an embedding for each of three input modalities: structure, full sequence, and a randomly selected subsequence of length 20 to 60 residues (the random length and starting position were chosen once per domain and fixed for all models). This process resulted in 95,088, 103,959, and 29,697 embeddings for Datasets S1–S3, respectively. To visualize the embeddings of a model as a two-dimensional map, we calculated a t-SNE projection (43) of the embeddings obtained for all three input modalities. Each point represents the embedding of one input modality of a single domain. We show the maps of Datasets S1 and S2, from the CLSS-sub and CLSS-full models, presented for comparison alongside maps obtained for three other state-of-the-art PLMs–ESM3 (mean of the per-residue embeddings) (25), ProstT5 (mean of per-residue embeddings) (39), and ProTrek (the first embedded vector) (30) (Fig. 2 and *SI Appendix*, Fig. S4 and S5–S14).

In both the CLSS-sub and CLSS-full models (Fig. 2 and *SI Appendix*, Fig. S4, first and second rows), the maps from structure and sequence inputs (second and third columns) are nearly identical, demonstrating that the models successfully coembed these two modalities. Moreover, both maps offer a well-organized representation of protein space, in that they place domains from the same architecture, and even the same structure class (e.g., ECOD all-α, all-β, α/β, and α + β domains), close to each other. In contrast, the projected embeddings of the sequence and structure modalities of the other PLMs are found in separate regions of their low-dimensional joint maps. ESM3 and ProstT5 do not aim to coembed these two modalities, and hence this separation is expected. ProTrek, however, was trained to coembed sequence and structure (albeit alongside embeddings of textual descriptions), and this separation suggests that the respective input modalities are organized differently in their full dimension. To examine the extent to which sequence and structure modalities are differently organized in the full dimension in ProTrek versus in the CLSS models, we adapted representation alignment measures from (44) and applied them to CLSS-sub, CLSS-full, and ProTrek. Briefly, we used the agreement between *k*-nearest neighbors (*k*NN) found among the sequence and structure embeddings, denoted SeqAgreeandStrAgree, respectively (see *Methods* for details). The agreement varies depending on the dataset and the size of the neighborhood *k*. The agreement is the lowest for ProTrek, with SeqAgreeandStrAgree ranging from 31 to 42%, compared to CLSS-sub with 35 to 71%, and CLSS-full with 65 to 89% (*SI Appendix*, Fig. S15 and Table S1).

### CLSS Recapitulates Expert-Curated Hierarchical Classifications.

**Fig. 3.**
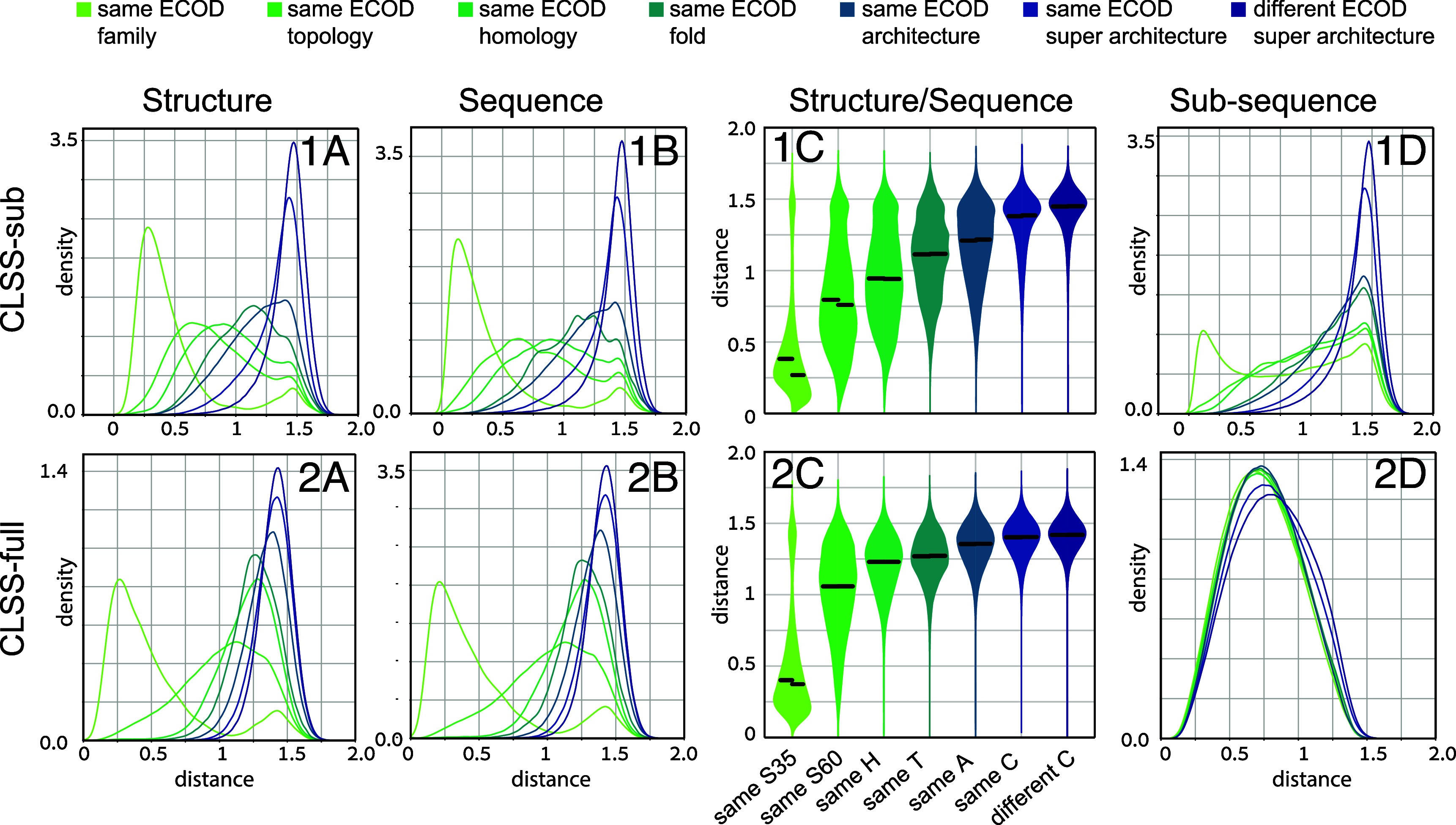
Estimated distance distributions between pairs of embeddings for the domains in Dataset S1. Each panel shows distance distributions of domain pairs sharing ECOD labels at the indicated level of the hierarchy. The color palette indicates the shared level in the ECOD hierarchy, from pairs with the same H-group label in light green through to pairs with a different structure class in purple. For the structure (*A*) and full sequence (*B*) modalities, the distances in both CLSS-sub (row 1) and CLSS-full (row 2) follow the ECOD hierarchy, with the domain pairs from the same H-group being closest. The violin plots pairing the distributions of distances in these two modalities (*C*) are largely symmetric, indicating that the distributions of these two modalities are similar. The CLSS-sub model captures a meaningful signal from subsequence inputs, as reflected by the distance distributions shifting to the right when considering domain pairs from increasingly distant ECOD levels (1D). The CLSS-full model does not capture subsequence information (2D).

To assess whether expert-curated hierarchical classifications, and structural similarity measures are reflected in the embeddings of CLSS and of other PLMs, the pairwise distances (in the full dimension) were calculated for domains of varying degrees of similarity. Distributions were then estimated using a Gaussian kernel density function. We grouped the domain pairs by decreasing similarity using three references. Specifically, for Dataset S1 (comprising ECOD domains), we used ECOD labels (Fig. 3 and *SI Appendix*, Fig. S16); ECOD classifies domains according to a five-level hierarchy, where the highest level of categorization is architecture, followed by possible homology (fold), homology, topology, and protein family. For Dataset S2 (comprising CATH domains), we used CATH labels, which classify domains according to class, architecture, topology, and homologous superfamily (*SI Appendix*, Fig. S17). Finally, for a set of 993 domains from the ProteinShake protein similarity dataset, we used the precalculated TM-align structure similarity scores (*SI Appendix*, Fig. S18). Even though hierarchical labels were never used during training [as in some other frameworks (45, 46)], we find that the distance distributions of the embeddings follow both the ECOD and CATH hierarchies, where domains from successively broader groupings have more and more dissimilar embeddings. We expect distance distributions in an informed embedding space to differ for different levels in the hierarchy (*e.g.*, same ECOD topology vs. same homology). We quantify the degree of difference using Jensen–Shannon divergence between the distributions (*SI Appendix*, Figs. S19 and S20). Comparing the separations of the embedding distance distributions from CLSS and other PLMs across all levels, we find that CLSS-full is the most informative, for both the sequence and structure input modalities. Finally, we note that the structure and sequence modalities yield virtually identical distance distributions for both CLSS-sub and CLSS-full, consistent with their embeddings being highly correlated (Fig. 3 and *SI Appendix*, Figs. S17 and S18, third column).

### CLSS Outperforms State-Of-The-Art PLMs on Downstream Classification Tasks.

Table 1 compares different PLMs’ performance on downstream classification tasks from the ProteinShake benchmark suite (40), Each task entails predicting, on the basis of the embedding calculated for an input domain (sequence or structure), a label corresponding to that domain in the SCOP (Structural Classification of Proteins) database. We used four types of tasks, corresponding to four categories of SCOP labels, respectively: family, superfamily, fold, or class. For each combination of model, input modality, and task, we report the model’s accuracy on the test set, based on the *k*NN of the model’s embeddings. Per machine learning best practices, we use the optimal *k* from the validation set. The train, validation, and test splits for the label prediction tasks were defined by the benchmark.

**Table 1.** Accuracy on SCOP label prediction from the dataset and three data splits by the ProteinShake benchmark (40), based on *k*NN searches of embeddings from the indicated model

		Sequence modality						Structure modality				
		CLSS-sub	CLSS-full	ESM2	ESM3	ProstT5	ProTrek	CLSS-sub	CLSS-full	ESM3	ProstT5	ProTrek
Structure Data Split	SCOP-FA	0.73	0.82	0.69	0.73	0.77	0.7	0.75	0.82	0.7	0.75	0.72
	SCOP-SF	0.79	**0.89**	0.8	0.82	0.86	0.75	0.83	**0.88**	0.8	0.85	0.8
	SCOP-CF	0.83	**0.91**	0.81	0.84	0.87	0.76	0.85	**0.91**	0.82	0.86	0.81
	SCOP-CL	0.95	**0.96**	0.9	0.93	0.93	0.84	0.95	**0.96**	0.92	0.95	0.91
Sequence data split	SCOP-FA	0.63	**0.75**	0.66	0.68	**0.75**	0.71	0.71	**0.76**	0.65	0.72	0.7
	SCOP-SF	0.73	**0.84**	0.73	0.77	**0.84**	0.77	0.81	**0.87**	0.76	0.82	0.77
	SCOP-CF	0.76	**0.86**	0.74	0.79	**0.86**	0.78	0.84	**0.88**	0.77	0.85	0.79
	SCOP-CL	0.93	**0.95**	0.88	0.9	0.94	0.9	0.95	**0.96**	0.9	0.93	0.91
Random data split	SCOP-FA	0.59	0.71	0.64	0.66	**0.73**	0.71	0.69	**0.73**	0.63	0.69	0.65
	SCOP-SF	0.68	0.8	0.73	0.74	**0.81**	0.76	0.78	**0.83**	0.74	0.78	0.74
	SCOP-CF	0.72	**0.82**	0.75	0.76	**0.82**	0.78	0.81	**0.86**	0.76	0.81	0.77
	SCOP-CL	0.89	**0.93**	0.87	0.88	**0.93**	0.89	0.92	**0.95**	0.89	0.9	0.9

Each task entailed predicting a specific category of SCOP label: family (SCOP-FA); superfamily (SCOP-SF); fold (SCOP-CF); or class (SCOP-CL), using an embedding generated by a single input modality (sequence or structure). For each combination of model, modality, and task, we report the accuracy on the test set when using the validation set optimal *k*. The top performing model in each modality/data split is marked in bold.

The benefit of coembedding the sequence and structure modalities using CL is evident when comparing the accuracies of the sequence modality embeddings of CLSS-full versus ESM2 (the latter being the initial pretraining weights of the CLSS sequence tower) and of the structure modality embeddings using CLSS-full versus ESM3 (which is modified by a trained adapter in CLSS). In both cases, we see an accuracy gain across all data splits and tasks. In other words, for any given input modality, the embedding produced by CLSS (a model trained on both sequence and structure data) facilitates greater accuracy in predicting the domain’s SCOP label. Overall, the CLSS-full model is the top performer in both sequence and structure modalities, demonstrating the benefit of coembedding the two modalities.

### CLSS Robustly Coembeds the Sequence and Structure of Metamorphic Domains.

Coembedding of proteins with similar sequences but dissimilar structures poses a unique challenge to contrastive learning: In the sequence modality, the embeddings should be near each other (due to sequence similarity) but in the structure modality the embeddings should be far apart (due to structural dissimilarity). To understand if and how CLSS resolves these conflicting signals, we mapped the CATH domains of Dataset S3, which, as noted above, are metamorphic, and as such are sequence-similar but structurally distinct. Encouragingly, the sequence and structure maps are generally similar to each other (*SI Appendix*, Figs. S21 and S22) and both modalities successfully group domains from the same architecture and superarchitecture, even though CATH labels were never used during training. To further study Dataset S3, we plotted the distance between the sequence and structure modalities for each of the pairs listed in (47) (*SI Appendix*, Fig. S23). Although the other PLMs compared here do not coembed modalities—and thus, one does not expect metamorphic proteins to present a challenge—the resulting groupings are far less pronounced (*SI Appendix*, Figs. S24–S26). Finally, a joint map of domains from Datasets S2 and S3 shows that the metamorphic CATH domains fall in different regions of the CATH domain space (*SI Appendix*, Fig. S27).

### CLSS Encodes the Context of Domain Subsequences.

The training of CLSS-sub included subsequences (see the Ablation and Hyperparameters subsection in the *Methods* for more details). We now evaluate how well the CLSS models embed domain subsequences as a third modality. The maps and distributions in Figs. 2 and 3 and *SI Appendix*, Figs. S2, S4–S14, and S16–S20 include embeddings for the subsequence modality. The first rows of Fig. 2 and of *SI Appendix*, Fig. S4 show maps of the embeddings for Datasets S1 and S2 from the CLSS-sub model. The maps obtained from the embeddings of the subsequence modality (panel 1*D*) are broadly similar to those obtained using the full sequence (panel 1*C*) and the full structure (panel 1*B*). Moreover, the embedding distance distributions for CLSS-sub from the subsequence modality (Fig. 3 and *SI Appendix*, Fig. S17, panel 1*D*) follow the same trend as the other modalities, with domains from broader groupings resulting in progressively more distant embeddings. The distances among subsequence embeddings even capture the separation among different levels of the hierarchies (*SI Appendix*, Figs. S19 and S20). In contrast, CLSS-full, ESM3, ProstT5, and ProTrek do not yield informative embeddings for the subsequence modality (Figs. 2 and 3 and *SI Appendix*, Figs. S4, S6–S9, and S11–S14). In each of the latter four cases, the subsequence embeddings occupy a separate region of the map, show convoluted patterns, and fail to recover hierarchical classifications.

There are cases where the embedding of a subsequence is far from the embeddings of the full domain from which it came, even for CLSS-sub, as can be seen in the stacked distance distributions in *SI Appendix*, Fig. S28. Often, these cases involve architectures containing both α and β elements—primarily mixed α + β and α/β (light green), α/β three-layered sandwich (dark green), and α/β barrel (green), and to a lesser extent α + β complex topology (brown) and α + β four layers (light brown). This result may reflect that α/β folds have significant fragment similarity between them, such that extracting a short fragment greatly diminishes information about the surrounding domain context.

### Cofactor Utilization Approximately Bisects the Protein Universe.

**Fig. 4.**
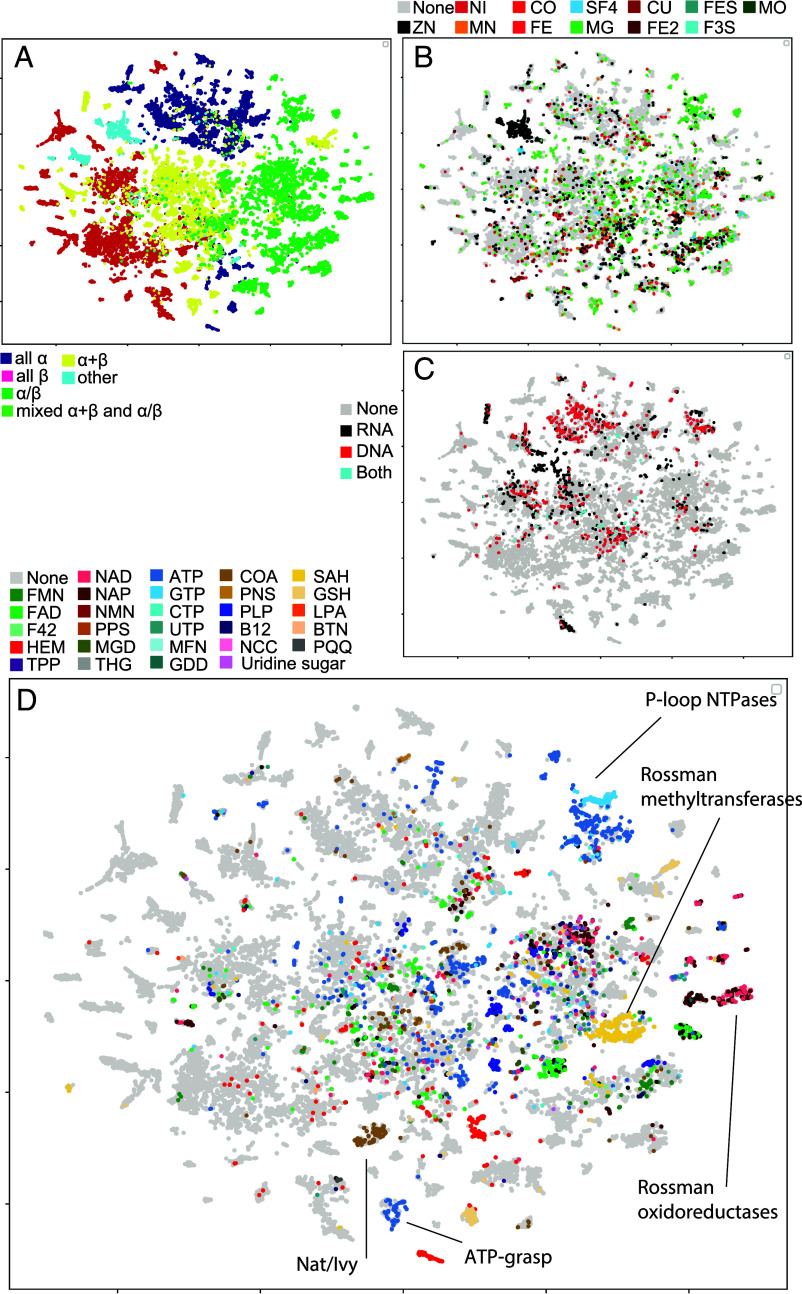
Cofactor utilization approximately bisects the protein universe. A map of the CLSS sequence embeddings of the ECOD (F40-develop291) dataset, colored by (*A*) ECOD class, (*B*) metal binding, (*C*) DNA/RNA binding, and (*D*) organic cofactor binding. Metal binding is found throughout. However, cofactor binding is enriched on the *Right* side of the map (corresponding to α/β and α + β ECOD classes) compared to the *Left* side of the map (corresponding to all-α and all-β classes).

Coloring the CLSS map based on protein structure class, metal associations, DNA/RNA binding, and cofactor associations (Fig. 4) reveals a striking split between domains with few cofactor associations (*Upper*-*Left* side of panel *D*) and those with strong cofactor associations (*Lower*-*Right* side of panel *D*). Comparing Fig. 4 *A* and *D*, we find that cofactor associations are very common among α/β and α + β domains, but much less common in α-rich domains, and very rare in β-rich domains. While the prevalence of α/β folds in metabolism has been noted previously (48–50), the map view afforded by CLSS brings this functional preference into sharp focus. It is tantalizing to hypothesize that the structure features of α/β and α + β folds, which often have α/β elements, confer a fundamental preference for cofactor binding. The special properties of βαβ fragments, and particularly of the N terminus of the α-helix and the loop that precedes it (11, 51, 52), may be the causal factor, though historical explanations cannot be ruled out.

The distribution of DNA and RNA binding, like cofactor binding, occupies restricted regions of the map, though with a less well-defined structural affinity than cofactor binding. Although some regions of overlap with cofactor binding occur (for example, among P-loop NTPases and Rossmanns) these two functionalities tend to occupy distinct regions of the map. The implication is that cofactor binding and nucleic acid binding likely favor different kinds of folds.

In contrast, metal utilization is sparsely distributed across almost all regions of the map (Fig. 4*B*). A few regions, however, have significant enrichment for a specific metal association. For example, zinc cations are strongly associated with a cluster of several X-groups annotated as having the “few secondary structure elements” architecture. A second cluster of domains from the same architecture, but that do not have strong associations with zinc, is close by, suggesting that CLSS near-hierarchically grouped these two clusters together (owing to their broad lack of secondary structure) and then split them according to the structure and sequence features associated with zinc binding. Other strong metal associations are in full agreement with expectation: Magnesium cation associations are enriched in the region of P-Loop NTPases, which have a conserved Mg^2+^ cation in the active site; and cupredoxins are strongly associated with copper ions.

### Limitations.

CLSS models were trained on domains, rather than complete protein chains, to reduce computational burden. The definition of what a domain is, however, varies (53), and CLSS models were trained on domains with ECOD-identified boundaries from a database that includes both experimentally solved and AF-predicted structures (42). This limitation does not suggest that the validations using ECOD and CATH labels suffer from data leakage of the labels, as the labels were not used during training. All validations were carried out with a domain-centric view of the protein universe; for example, the *SeqAgree* and *StrAgree* measures compared neighborhoods of full domain embeddings. Thus, CLSS models are best suited for use with domains that have ECOD-defined boundaries, as these are from a similar distribution to the one the model trained on. When using our models, we recommend decomposing a protein chain into domains and considering the embeddings of the latter.

We generated visualizations of the 32-dimensional latent space of CLSS using t-SNE with a perplexity of 30. Dimensionality reduction may distort relationships between domains. One may consider alternative perplexity values or other methods such as UMAP.

### The CLSS Viewer.

We provide a web interface for exploring a t-SNE projection of the CLSS embeddings of almost all ECOD domains from the ECOD-AF2 F100 develop292 dataset (https://gabiaxel.github.io/clss-viewer/). Individual domains or groups of domains can be searched for by their domain identifier (e.g., “e6tjeA1”), evolutionary grouping (e.g., “207,” “207.1”), and partial name (e.g., “lectin”). When an area of interest on the plot is selected, the list of associated domains, along with their ECOD classifications and (x, y) coordinates, are displayed. To facilitate data sharing, the URL is updated to stably reflect the current selection state. In addition, selected domain data can be downloaded as a CSV file for offline analysis. Fig. 4, S30, and S31 demonstrate the usefulness of the viewer.

## Discussion

We introduced CLSS, a self-supervised sequence–structure coembedding modeling approach, and demonstrated its use in mapping the protein universe. The goal of mapping is to construct a coherent organization of protein space for evolutionary inference and classification tasks; thus, mapping serves as an essential counterpart to models optimized for structure prediction and sequence generation. Given a protein domain sequence or structure (including a predicted AF structure), CLSS positions this domain next to similar or related domains in the latent space (and in resulting maps). Notably, similarity in this context relates to both sequence and structure and, in the case of CLSS-sub, even subsequences can be embedded. The CLSS viewer provides an easy-to-use, interactive interface for protein space exploration.

CLSS-full is a model that can successfully coembed protein sequence and structure (Fig. 2 *B* and *C* and *SI Appendix*, Fig. S15). This outcome shows that sequence and structure spaces can be organized in a compatible way, despite their complicated many-to-many relationships. CLSS-full learned this organization using a contrastive loss function that pushed the embeddings from each input modality together, allowing them to jointly construct the learned embedding space. Thus, while embeddings are still calculated from either a sequence or structure input, the result reflects information derived from both modalities. In the case of sequence inputs, coembedding incorporates a learned structural signal while avoiding the computational cost of predicting and processing structures. As hypothesized, this richer signal performs better on downstream tasks. More broadly, our results support the growing evidence that learning a shared embedding space across complementary biological modalities can produce more informative representations for downstream applications [e.g., as shown in ConPlex (54)]. It is noteworthy that CLSS embeddings are only 32-dimensional, much lower than a typical LM. That they are informative nevertheless underscores the intricate evolutionary links between protein sequence, structure, and function.

Maps, created with dimensionality reduction tools, visualize the latent representations, or embeddings, learned by CLSS and other models. Our focus, thus, is on these nonmodified vectors, and it is their utility that we evaluate in the downstream tasks. The modality-based separations in maps generated using ProTrek suggest that this model organizes the embeddings of different modalities differently in the full dimension. This separation is unlikely to be a dimensionality-reduction artifact, since the dimensionality reduction tool is unaware of the modality of the input embeddings. kNN searches in the full dimension support this interpretation, indicating a lack of coembedding by ProTrek. In comparison, CLSS models produce consistent embeddings irrespective of data modality.

The agreement of CLSS maps with the hierarchical labels used in the CATH and ECOD databases is striking, especially in light of the model’s self-supervised training, which did not have access to label information (e.g., in contrast to refs. 45, 46, 55). The fact that CLSS maps capture documented similarities without being exposed to that information during training suggests that the distances among other embedded domains are also reasonable. Accordingly, cases where hierarchical classifications and CLSS disagree could highlight events warranting structural exploration, e.g., connections between different folds and possible examples of structure divergence (see additional discussion and examples in the Supplementary Text). More broadly, considering CLSS-produced maps in combination with expert knowledge of diverse facets of the protein universe (23, 25–28, 33, 34, 56) provides opportunities to reveal global patterns in protein space. Maps can be particularly illuminating when overlaid with functional properties, such as ligand binding, as exemplified here. In short, CLSS offers a new, AI-based perspective on the continuous, malleable nature of the protein universe, a long-pursued scientific challenge (1).

CLSS-sub embeds subsequences next to the embedding of their full structure and sequence. This is a unique PLM in that it is trained with the goal of including protein subsequences (or fragments), and, indeed, the subsequence signal in all other models is not meaningful. The motivation to embed subsequences stems from their importance in protein evolution and design. Protein subdomain fragments are extensively reused across the protein universe, with some suspected to have founder roles in the first full domains (8–11). Early protein structure prediction approaches based on protein threading (57) suggested that the compatibility between a sequence and a (potentially much larger) structure is to some extent predictable (58–60). However, the many-to-many relationships with the far larger and less informative space of subsequences complicate matters further. Thus, it was not clear a priori that it would be possible to train a model that coembeds all three modalities. Our analysis shows that CLSS-sub coembeds the full sequence and structure modalities less well compared to CLSS-full. Nonetheless, CLSS-sub successfully coembeds all three modalities, full sequences, subsequences, and full structure, with each modality yielding similar maps of protein space.

The CLSS models include several practical improvements: With only 36 M trainable parameters, these models are considerably smaller than other state-of-the-art models, even those considered lean (61). Thus, training purpose-built models with CLSS-like architectures is possible with only modest computational resources. In addition, CLSS models use a compact embedding space of only 32 dimensions (62), facilitating data storage and searching (63). These design choices provide an alternative view on the future of PLMs, with a better bias–variance tradeoff that creates more efficient and compact models (64).

We anticipate that CLSS, or models like it, will be a valuable resource for molecular biologists. Ultimately, a unified sequence–structure embedding space will create new possibilities for database searching, sequence alignment, protein engineering, and evolutionary trajectory analysis.

## Methods

### CLSS Architecture.

Fig. 1 illustrates the architecture of CLSS. Our contrastive model has a two-tower architecture: The sequence tower is a trainable model with an architecture and initial weights like the ESM2 35 M-parameter model (26); the structure tower is a frozen ESM3 (2) model with 1.4B parameters. Given a protein (or domain) with N residues, the sequence and structure towers infer a matrix of size N×K, where K is a model-dependent constant (480 for the sequence tower and 1536 for ESM3). Taking the mean over the matrix columns for each input results in vectors of length 480 and 1536, respectively. Finally, an adapter (a single linear layer) of size K×dim_size is appended at the end of each model, followed by L2 normalization. For every sequence–structure pair, CLSS outputs two vectors of length dim_size.

### Datasets.

Our training/validation dataset comprises 1 million randomly sampled (unlabeled) domains from the publicly accessible ECOD domain classification of experimentally determined and AlphaFold 2 predicted structures (42). CLSS uses ESM3’s ProteinChain API and disregards domains that have PDB files the API failed to open. Following machine learning best practices, we randomly split the dataset into a training set with 950,000 domains and a validation set with the remaining 50,000 domains. We present maps of three datasets: Dataset S1 comprises 31,696 ECOD domains taken from the 109 most-populated protein evolutionary lineages (ECOD X-group), spanning 16 architectures (*SI Appendix*, Table S1). Dataset S2 comprises 34,653 CATH (S40) domains, and Dataset S3 comprises 9,899 CATH domains from Røgen’s set of sequence-similar but topologically distinct domain pairs (36) (*SI Appendix*, Table S1). We also rely on the datasets of the ProteinShake (40) benchmark. The CLSS viewer shows all domains in the ECOD-AF2 develop292 F100 dataset with a valid PDB file (approximately 1.81 million) except for 95 domains with missing or corrupt PDB files that our download pipeline failed to fix. In cases where the provided PDB files were invalid, we applied the PDBFixer python library. In cases where the provided PDB files were empty, we used the PDB file from the ECOD-PDB dataset instead and, if that file was empty as well, we used the relevant PDB file from RCSB and cut it manually according to the domain’s start and end residues as listed on ECOD-PDB develop291.

### Training Process.

In each training step, 1440 (8 minibatches, each of size 180) domains (illustrated in Fig. 1*B* with a batch of size 4) were randomly selected from the full training dataset. Each minibatch was assigned to a different GPU. For each domain, we sampled a random subsequence of length *l,* starting at position *s* (highlighted in Fig. 1*B* as boxes over the sequence). s and l were sampled at random from the uniform distributions over the ranges [0, *domain_length – min_l + 1*] and *[min_l, domain_length - s + 1]*, respectively, where *min_l* is a parameter fixed per model (see Ablation Studies and Hyperparameter Search for more details on the impact of the choice of *min_l* on the trained model). We then calculated the sequence-tower embeddings for these subsequences and projected them to a normalized vector of the embedding dimension (annotated in Fig. 1*B* as $Se_{1},\cdots ,Se_{4}$). We also calculated the structure-tower embeddings for the full domains based on the frozen ESM3 model followed by an adapter to project to a normalized vector of the embedding dimension (annotated as $St_{1},\cdots ,St_{4}$). To compute our loss function, we calculated the dot-product matrix of all subsequence and structure embeddings in each minibatch and divide the entire matrix by a temperature scalar τ. Then, we apply the SoftMax function to each row in the matrix, and calculate the Cross-Entropy loss on the resulting distributions, while labeling values on the diagonal (corresponding to subsequences and structures of the same domain) as the “correct” class.

$$L_{\text{contrastive}}\left(E_{\mathrm{seq}},E_{\mathrm{str}}\right)=\text{CE}\left(\text{SoftMax}\left(\frac{E_{\mathrm{seq}}\cdot E_{\mathrm{str}}^{T}}{\tau }\right)\right).$$

Our contrastive objective maximizes the dot product between the structure and subsequence embeddings from the same domain (marked in green), while minimizing it between different domains (marked in gray). Overall, we trained the network for a fixed number of 80 epochs on 8 NVIDIA A100 (40 GB) GPUs using the PyTorch Lightning framework and DDPstrategy for approximately 4.5 d. *SI Appendix*, Fig. S29 shows training and validation set losses for the different trained models.

The training process updated all parameters of the sequence tower in the model and the adapter networks of both towers, while keeping the parameters of the ESM3 network frozen. This design implemented our goal of injecting structure information into the sequence tower. Overall, the number of trained parameters in our network was only ~ 36 M, slightly more than the small ESM2 (35 M) model, and dramatically less than ProstT5 (3B), ESM3 (1.4B), and ProTrek (930M).

Our evaluation was inspired by CLIP: The coembedding was trained on the pairing of extensive data, followed by evaluation on labeled data. In CLSS, the pairing is between the full/subsequences and the structures of protein domains in large datasets. Importantly, neither ECOD nor CATH labels were used during training in any way. Consequently, analysis with respect to ECOD and CATH labels cannot suffer from data leakage. By randomly extracting the training and validation domain datasets from the same distribution of domains—as opposed to enforcing that these datasets have minimal overlap—we ensure that the learned latent space is preferentially organized by the most ubiquitous domain families. For the goal of visualizing a meaningful representation of protein space, one must train on an exhaustive dataset representing it, as do all of the methods compared here—ProstT5, ESM3, ProTrek, and the CLSS models.

### *SeqAgree/StrAgree* Measures of Coembedding.

We adapt from (44) two self-consistency measures, denoted SeqAgree/StrAgree, to evaluate the consistency of the coembedding of the full sequences and structures by different models in a database (DB) of domains. For each protein p∈DB, we denote the sequence and structure embeddings by ${\mathrm{em}}_{\mathrm{seq}}\left(p\right)$ and ${\mathrm{em}}_{\mathrm{str}}\left(p\right)$, and the modality-dependent embedding datasets by ${\mathrm{DB}}_{\mathrm{seqs}}=\left\{{\mathrm{em}}_{\mathrm{seq}}\left(p\right)\right|p\in DB\}$ and $DB_{\mathrm{strs}}=\left\{em_{\mathrm{str}}\left(p\right)\right|p\in DB\}$. For a query protein *q*, we denote the k-sized neighborhoods of its nearest sequence embeddings by $knn_{{\mathrm{DB}}_{\mathrm{seqs}}}\left(em_{\mathrm{seq}}\left(q\right)\right)$ and $knn_{{\mathrm{DB}}_{\mathrm{strs}}}\left(em_{\mathrm{seq}}\left(q\right)\right)$. We denote the size of the intersection of these two neighborhoods by $SeqAgree\left(q\right)=\left|knn_{CATH_{\mathrm{seqs}}}\left(em_{\mathrm{seq}}\left(q\right)\right)\cap knn_{CATH_{\mathrm{strs}}}\left(em_{\mathrm{seq}}\left(q\right)\right)\right|$.

Similarly, the neighborhood agreement centered on the structure embedding is $StrAgree\left(q\right)=\left|knn_{CATH_{\mathrm{strs}}}\left(em_{\mathrm{str}}\left(q\right)\right)\cap knn_{CATH_{\mathrm{seqs}}}\left(em_{\mathrm{str}}\left(q\right)\right)\right|$.

Notice that for every q and every k, $0\leq SeqAgree\left(q\right),StrAgree\left(q\right)\leq k$. To evaluate the coembedding, we consider the values of *SeqAgree/StrAgree* for all proteins in the DB, where higher agreement indicates better coembedding.

### Ablation Studies and Hyperparameter Search.

We trained multiple CLSS models with similar architectures, varying the sizes of the adapters to control the embedding dimension (considering 8, 16, 32, 64, and 128). We also varied the minimum length, denoted *min_l*, of the random subsequences sampled from each full domain sequence (considering 1, 10, 15, 20, 25, and 30 residues), as well as training using the full-length sequences only, without any subsequence sampling. We also considered multiple values for the temperature scalar: 0.25, 0.5, 1, and 2.

First, we evaluated the trained models by the distributions of distances between the sequence, structure, and subsequence embeddings for each domain in the held-out validation set of 50,000 ECOD domains (*SI Appendix*, Fig. S1). We found that increasing the size of the adapter networks yielded slightly higher distances between the different modalities (panels *A*1–*A*3). Training with a higher temperature parameter led to lower distances between every pair of modalities (panels *B*1–*B*3), and the difference is most noticeable in the distances between the full sequence and structure modalities (panel *B*2). Training on shorter subsequences resulted in slightly larger distances between the sequence and structure modalities (panel *C*2), but significantly smaller distances between the subsequence modality to both the sequence and structure modalities (panels *C*1 and *C*3). Training only on full sequences (panels *C*1–*C*3, light blue) yielded the shortest distances between the sequence and structure modalities, but had the opposite effect with respect to the subsequence modality, for which distance distributions to both the sequence and structure modalities were shifted significantly toward greater values.

We also evaluated the coembedding of the full sequence/structure modalities on the validation set for the CLSS models trained with different parameters, using *SeqAgree* (*SI Appendix*, Fig. S2) and *StrAgree* (*SI Appendix*, Fig. S3). The most coherent model when varying the dimension size of the embedding is 32 (panels *A*1–*A*3, black). Interestingly, the models trained with a higher temperature parameter have less coherent neighborhoods (*SI Appendix*, Figs. S2 and S3, panels *B*1–*B*3), even though the pairwise sequence–structure embedding distance is lower (*SI Appendix*, Fig. S1, panel *B*2). When considering the coherence of the full sequence–structure modalities, we found that it increased when we only included longer subsequences during training, and it was greatest when training only on the full sequences (*SI Appendix*, Figs. S2 and S3, panels *C*1–*C*3, light blue).

### Embedding Visualizations.

To create the t-SNE plots in Figs. 2 and 4 and *SI Appendix*, Figs. S4–S14, and in the CLSS viewer, we used 1,000 iterations and a perplexity value of 30. We visualized the probability density function of the distances between embeddings using the Gaussian kernel density estimator (gaussian_kde routines) from the scipy.stats package.

### Cofactor, Metal, RNA/DNA-Domain Associations.

Associations between domains and ligands, either metals or organic cofactors, were extracted from the ECOD-PDB-development-291 flat file (last column). Associations were calculated using a 4 Å ligand-domain distance cutoff. Common cofactors, as well as their derivatives—including reactant and product forms of the same cofactor (e.g., ATP and ADP), common derivatives (e.g., CoA and acetyl-CoA), and common nonreactive analogs (e.g., phosphoaminophosphonic acid-adenylate ester for ATP)—were grouped together (*SI Appendix*, Table S2). For clarity, domains that bind two or more ligands of the same type (less than 5% of domains that bind a ligand) were colored for just one of the ligands. For this set, to identify DNA/RNA binding, we downloaded the full protein files, extracted the DNA/RNA structures and tested whether any atom in the ECOD domain lies within 4 Å of an atom in the DNA/RNA structures.

### Similarities Between ProteinShake and CLSS Training Proteins.

We used BLAST to identify similarities. To this end, we created a BLAST database from the sequences of the proteins in the training and validation sets and queried it (using blastp) with the sequences of all ProteinShake proteins. For each query, we extracted the following values for its best alignment (in terms of lowest E-value): percent identity, alignment length, and the alignment length as a percent of the query length. *SI Appendix*, Fig. S32 shows the distribution of similarities found for the ProteinShake dataset of 993 proteins.

## Supplementary Material

Appendix 01 (PDF)

Dataset S01 (XLSX)

Dataset S02 (XLSX)

## Data Availability

Code data have been deposited in GitHub (https://github.com/guyyanai/CLSS) (65).
