# Supplementary material for: Contrastive learning unites sequence and structure in a global representation of protein space
Source: Proc Natl Acad Sci U S A. 2026 Aug 3;123(32):e2532702123. doi: 10.1073/pnas.2532702123 (PMC13462929; doi:10.1073/pnas.2532702123)
Supplement: Supplementary file 1 [file pnas.2532702123.sapp.pdf]

Contrastive learning unites sequence and structure in a global representation of protein space

Guy Yanai[1], Gabriel Axel[2], Liam M. Longo[3,4]* Nir Ben-Tal[2]*, Rachel Kolodny[1]*

[1]Department of Computer Science, University of Haifa, Haifa, Israel

[2]School of Neurobiology, Biochemistry and Biophysics, George S. Wise Faculty of Life Sciences, Tel Aviv University, Ramat Aviv, Israel

[3]Earth-Life Science Institute, Institute of Science Tokyo, Tokyo, Japan

[4]Blue Marble Space Institute of Science, Seattle, USA

*To whom correspondence should be addressed. E-mail: llongo@elsi.jp; bental@tauex.tau.ac.il; trachel@cs.haifa.ac.il

Supplementary figures

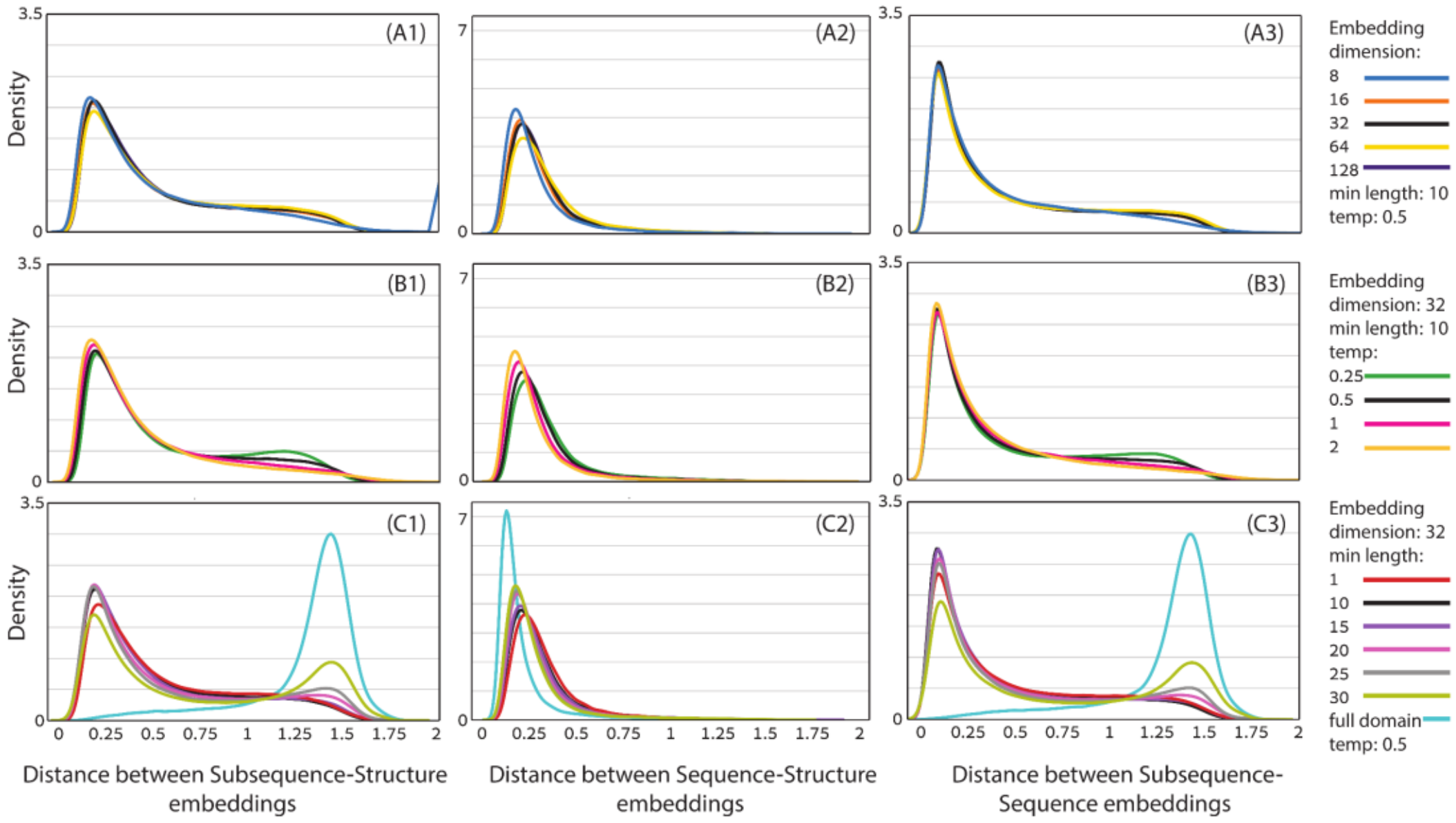

Figure S1: Distributions of distances, computed by different models, between embeddings from two modalities, for all domain in the validation set. The columns vary the pairs of modalities shown (1: subsequence-structure, 2: sequence-structure, 3: subsequence-sequence). The rows show the parameter that we vary among the considered models: (A: the embedding dimension, B: the temperature, C: the min_l variable). We see that the obtained distances are very similar when considering different dimensions for the latent space, higher temperatures lead to the models pushing the embeddings of the same domain in different modalities closer together; and support of subsequences is only meaningful when training with subsequences (including diminished support even when using min_l=30).

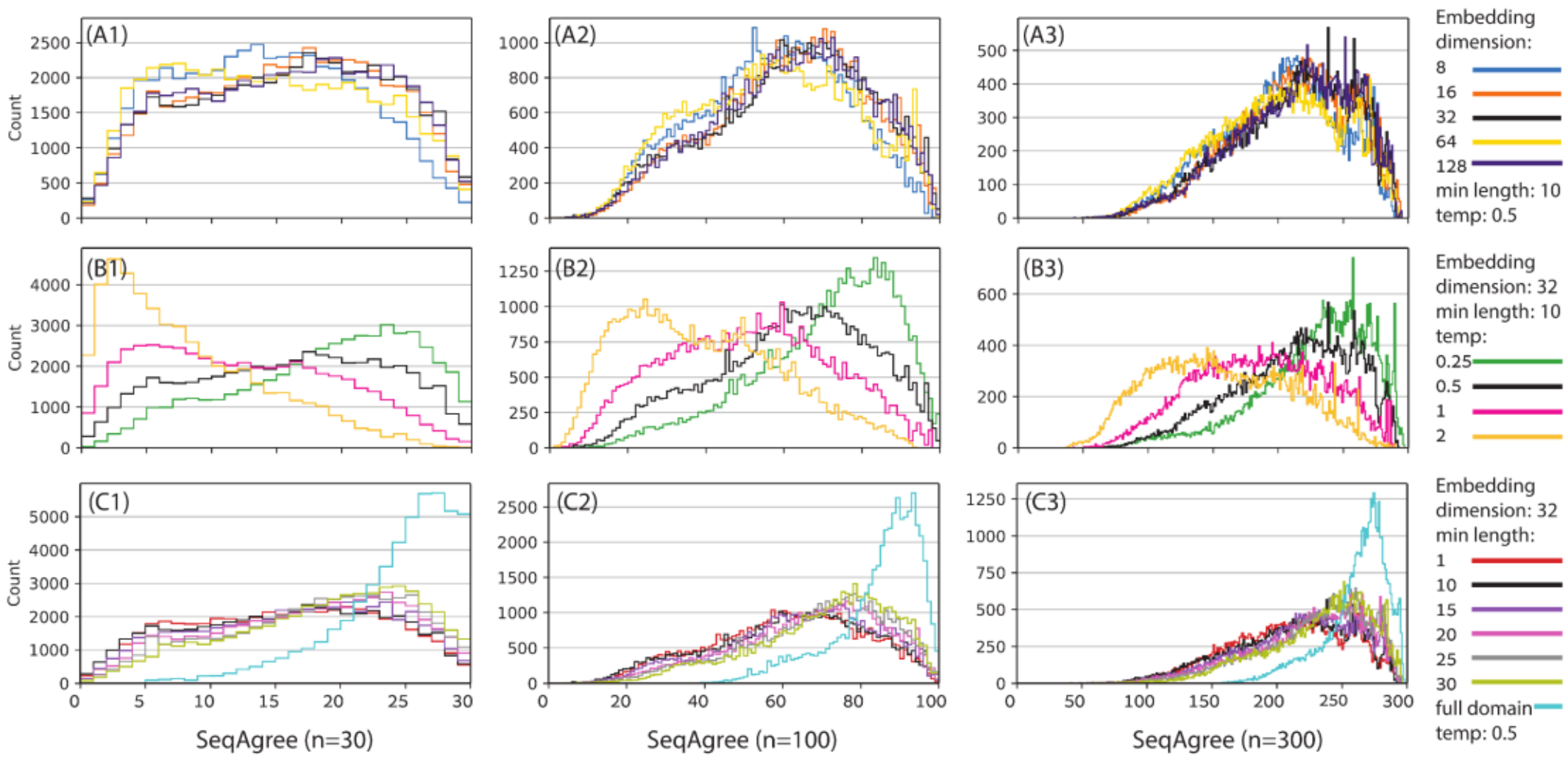

Figure S2: Distributions of SeqAgree values to evaluate the coherence of the co-embedding on the validation set for CLSS models trained with different parameters. Here, we consider the coherence centered around the sequence embeddings; for a coherence centered around the structure embeddings, see Figure S26. The columns vary the sizes of the neighborhoods (30, 100, 300 nearest neighbors), and in all cases, the distributions of more coherent models shift to the right. The rows show the parameter that we vary among the considered models: (A: the embedding dimension, B: the temperature, C: the min_l variable). We see that coherence is generally similar when across different dimensions for the latent space. As for the temperatures, we see that higher temperatures lead to less coherent models. Also, the support of subsequences leads to diminished performance in terms of the coherence of the modalities of full sequences and structures.

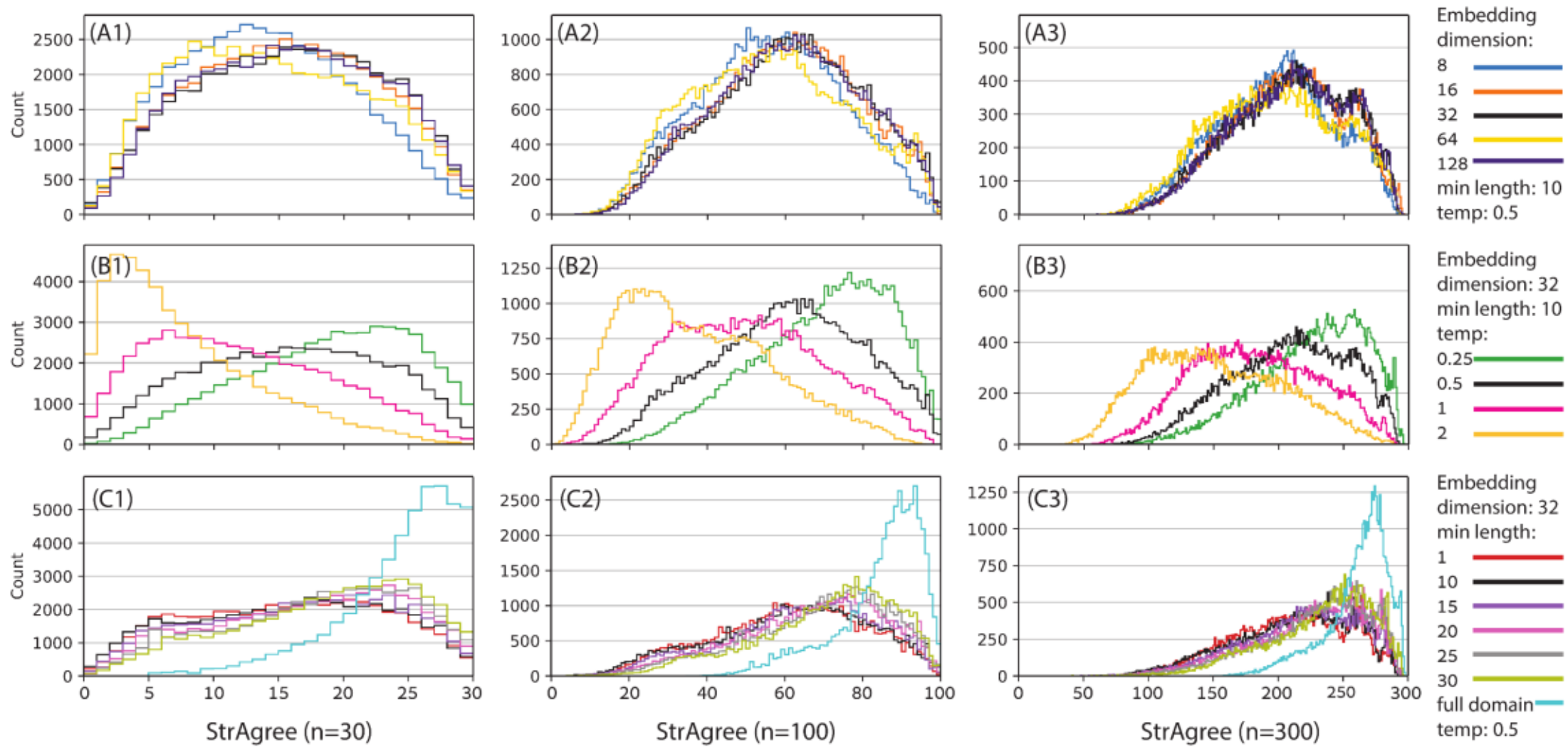

Figure S3: Distributions of StrAgree values to evaluate the coherence of the co-embedding on the validation set for CLSS models trained with different parameters. This is similar to Figure S2, only we consider the coherence centered around the structure embeddings. The columns vary the sizes of the neighborhoods (30, 100, 300 nearest neighbors), and in all cases, the distributions of more coherent models shift to the right. The rows show the parameter that we vary among the considered models: (A: the embedding dimension, B: the temperature, C: the min_l variable). We see that coherence is generally similar across different dimensions for the latent space. As for the temperatures, we see that higher temperatures lead to less coherent models. Also, the support of subsequences leads to diminished performance in terms of the coherence of the modalities of full sequences and structures.

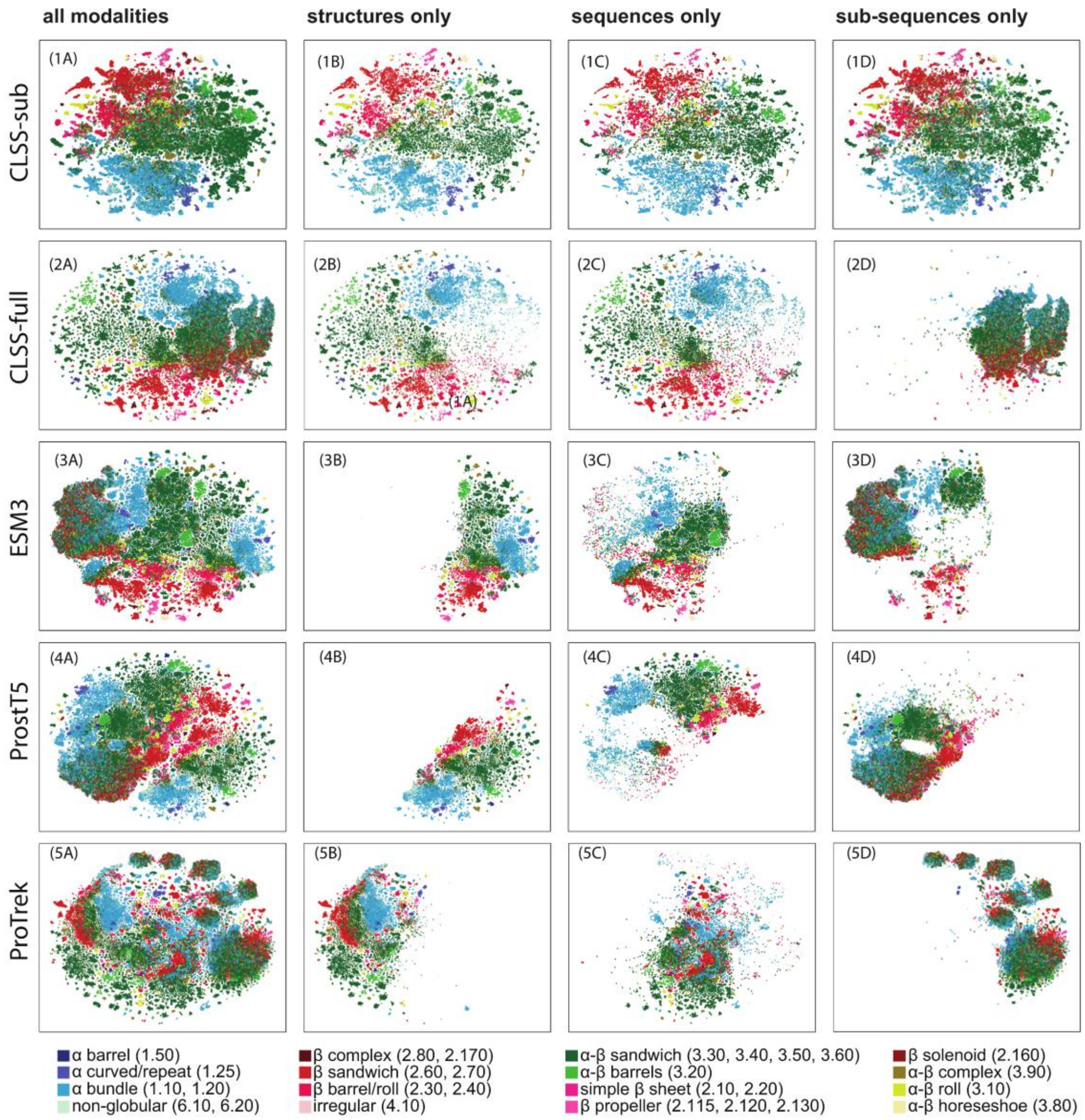

Figure S4: (equivalent to Figure 2 of the main text): Comparison of maps generated for embeddings of CATH domains (Dataset 2) with the PLMs (from top to bottom): CLSS-sub, CLSS-full, ESM3, ProstT5, and ProTrek. In each row, the maps (left to right) are an overlay of all three modalities, followed by visualization of only a single modality: structure, sequence, and sub-sequences. ***Figures S8-S12*** *show enlarged views of the embeddings of each model. The figure is similar to Figure 2 of the main manuscript, with a different dataset, and shares the same properties: in ESM3, ProstT5, and ProTrek, the embeddings of different modalities occupy distinct regions of the map. In CLSS-full, the full sequences and structure modalities overlap and have similar maps, but the subsequence modality occupies a separate region of the space. In CLSS-sub, all three modalities create generally similar maps.*

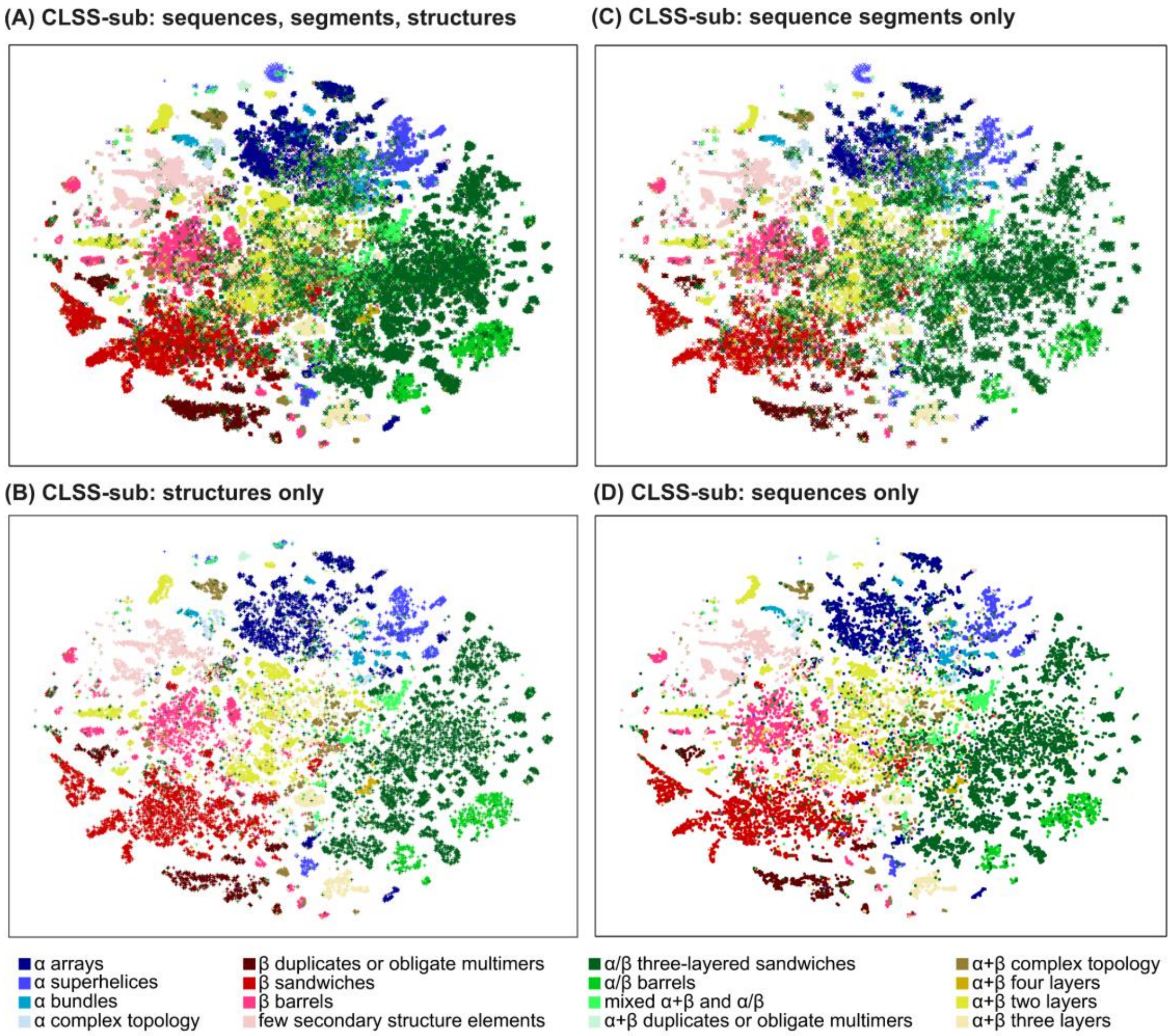

Figure S5: CLSS-sub embedding maps of ECOD domains (Dataset 1) a larger version of Figure 2, first row in the main manuscript. For each domain, we calculate the embeddings by three modalities – structure, sequence, and a random sequence segment – and then compute a t-SNE projection of the dataset of all embeddings. Each point represents one of the modalities of a domain colored according to the label of its ECOD architecture. (A) Map of all three modalities. Sequences are marked by circles, structures by '+', and random sequence segments by 'x'. For clarity we show only (B) structure embeddings, (C) sequence segment embeddings, (D) sequence embeddings. We find that the maps of all three modalities are very similar to each other, with the sequence (D) and structure (B) embeddings being the closest. The global organization of the CLSS-sub embedding space positions domains with the same ECOD architecture, and even the same structure class, near each other.

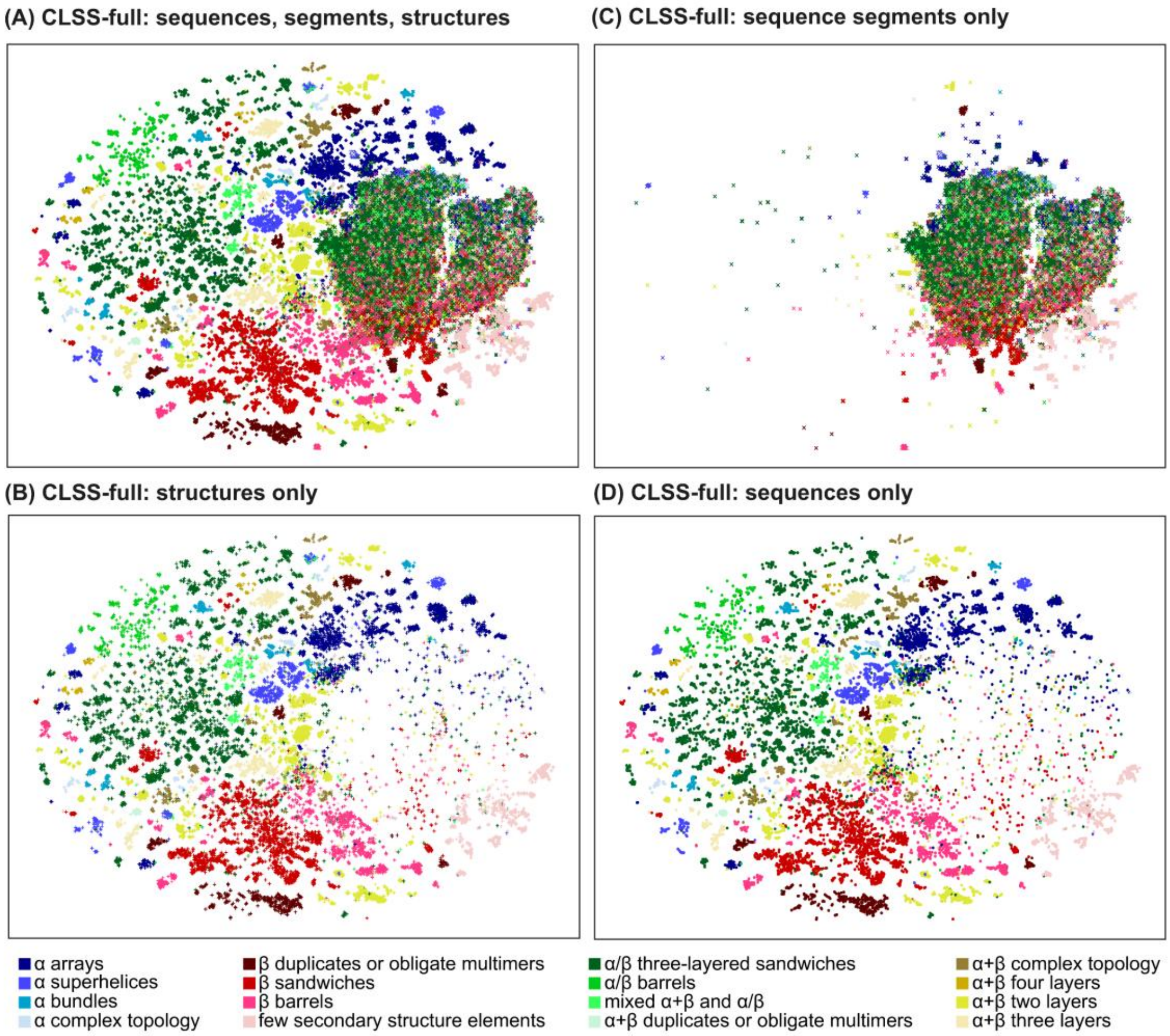

Figure S6: CLSS-full embedding maps of ECOD domains (Dataset 1): a larger version of Figure 2, second row in the main manuscript. For each domain, we calculate the embeddings by three modalities – structure, sequence, and a random sequence segment – and then compute a t-SNE projection of the dataset of all embeddings. Each point represents one of the modalities of a domain colored according to the label of its ECOD architecture. (A) Map of all three modalities. Sequences are marked by circles, structures by '+', and random sequence segments by 'x'. For clarity we show only (B) structure embeddings, (C) sequence segment embeddings, (D) sequence embeddings. We find that the maps of all three modalities are very similar to each other, with the sequence (D) and structure (B) embeddings being the closest. CLSS-full maps the subsequence modality to a separate region of the latent space from that of the full sequence and structure modalities, and this modality if far less organized compared to the full sequence and structure modalities. For the full sequence and structure modalities, their maps are generally similar to each other, and similarly position domains with the same ECOD architecture, and even the same structure class, near each other.

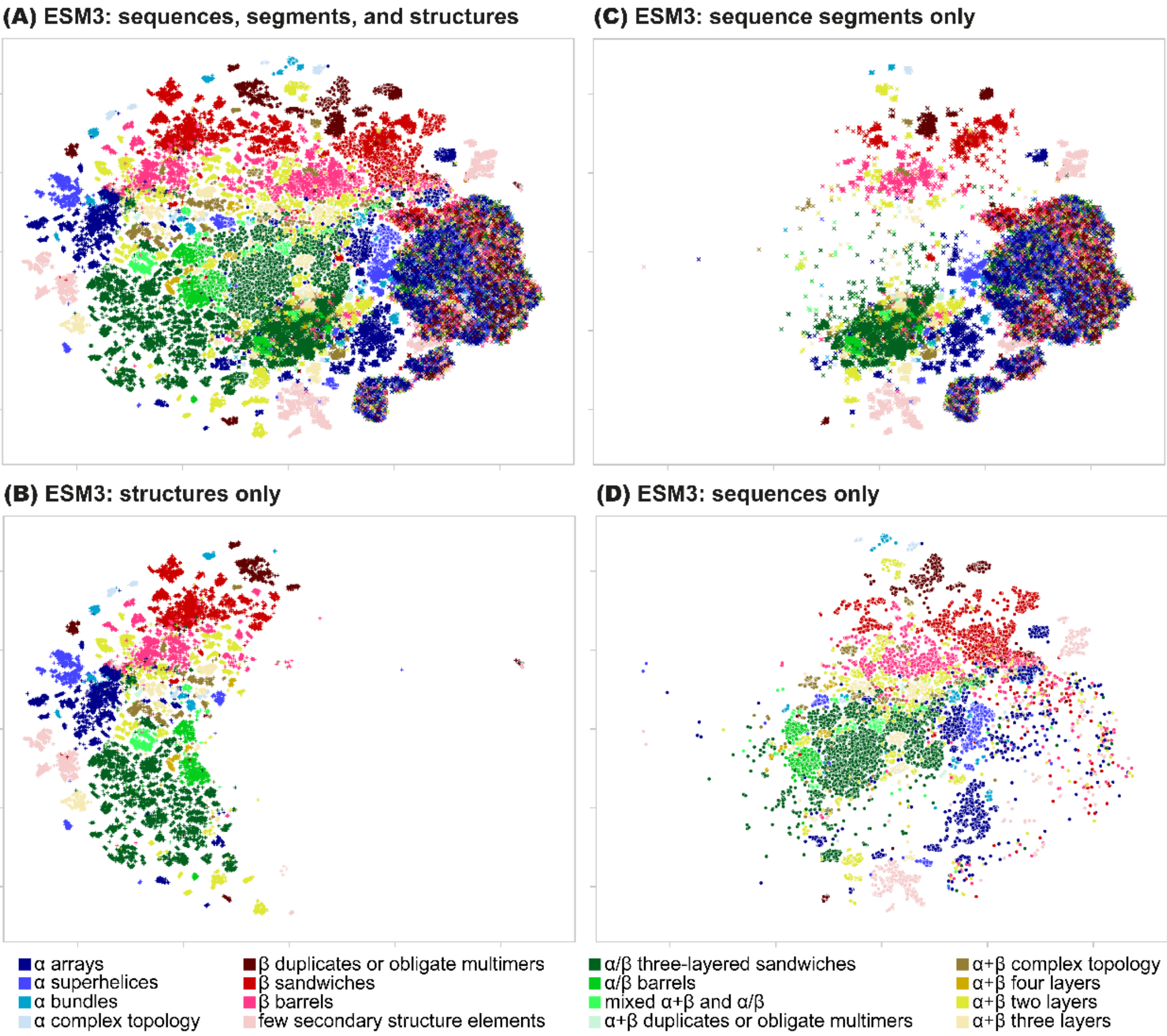

Figure S7: ESM3 maps of ECOD domains (Dataset 1) – a larger version of Figure 2, third row in the main manuscript. For each domain, we calculate the ESM3 embeddings by the structure, sequence, and a random sequence segment. Each point represents one of the modalities of a domain colored according to the label of its ECOD architecture. In this enlarged view we see that the structure modality is best aligned with the ECOD labels, the sequence modality less so, and the signal of the embeddings of the random sequence segment is very weak.

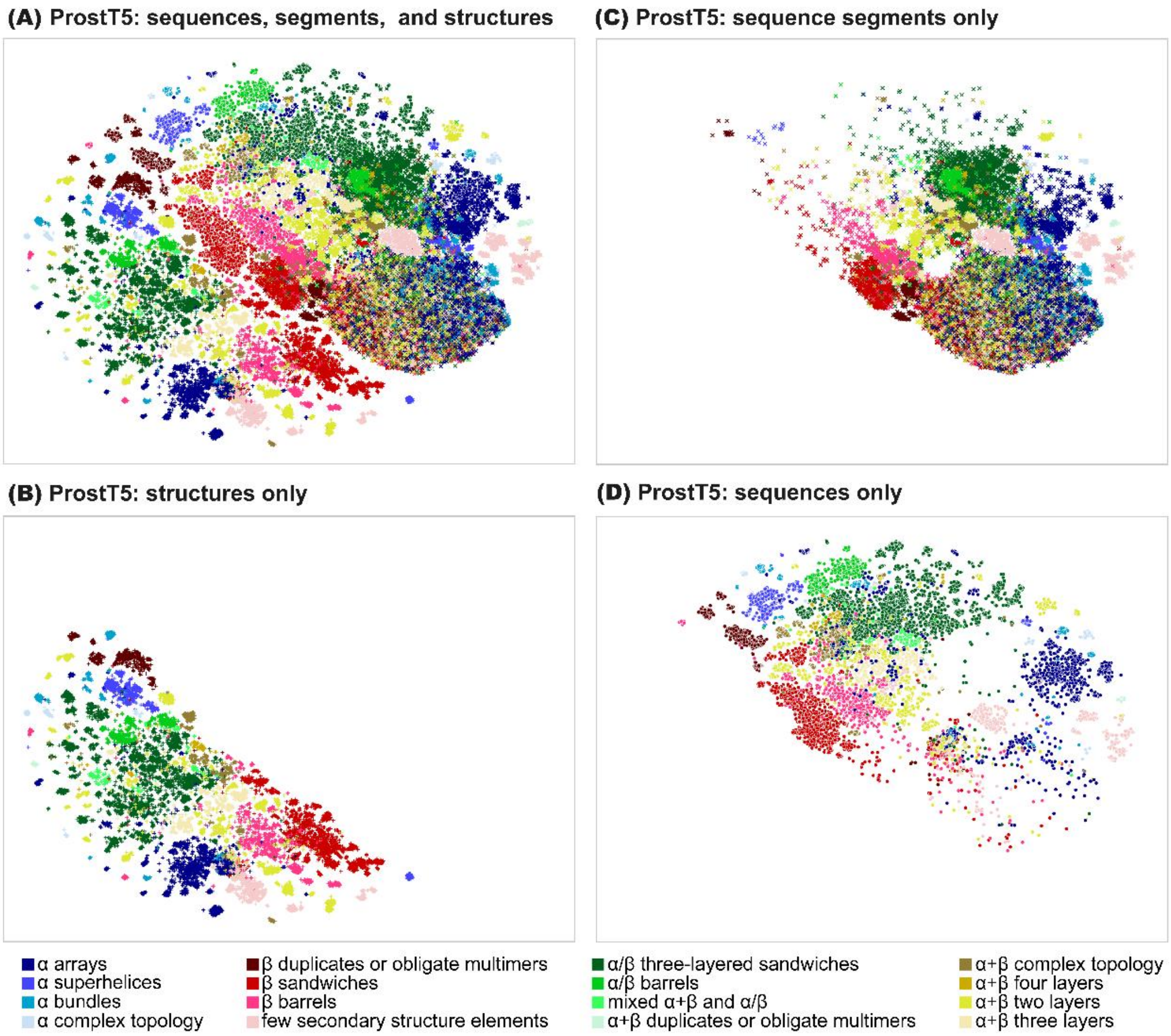

Figure S8: ProstT5 maps of ECOD domains (Dataset 1) – a larger version of Figure 2, forth row in the main manuscript. For each domain, we calculate the ProstT5 embeddings by the structure, sequence, and a random sequence segment. Each point represents one of the modalities of a domain colored according to the label of its ECOD architecture. In this enlarged view we see that both the structure modality and sequence modality capture the ECOD labels well, and the subsequence modality far less so.

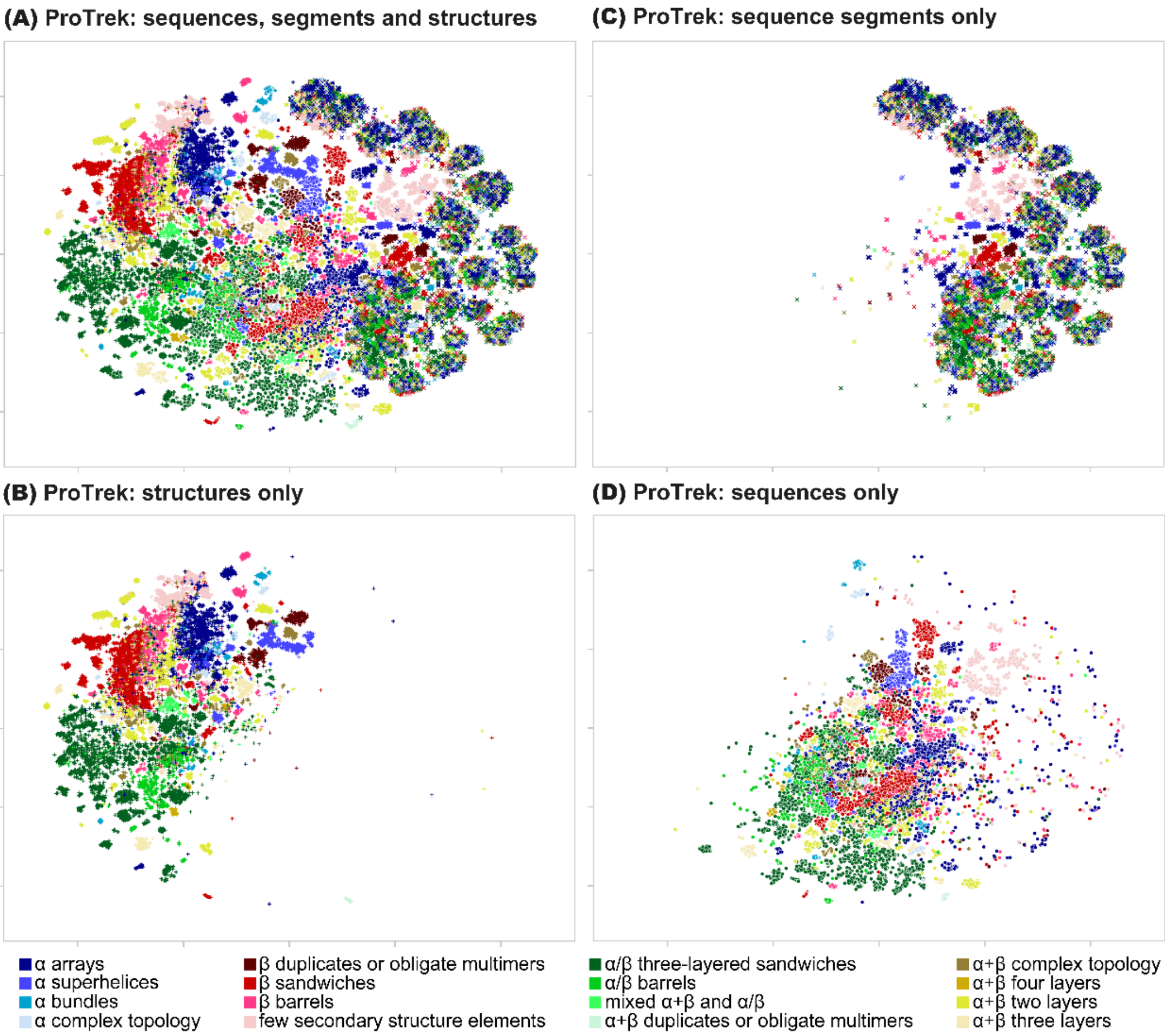

Figure S9: ProTrek maps of ECOD domains (Dataset 1) – a larger version of Figure 2, fifth row in the main manuscript. For each domain, we calculate the ProTrek embeddings by the structure, sequence, and a random sequence segment. Each point represents one of the modalities of a domain colored according to the label of its ECOD architecture. In this enlarged view we see that the agreement of the structure modality with the ECOD labels is the strongest, far less so for the sequence modality, and when considering the subsequence modality there is virtually no signal. Also, the embeddings of the sequences and the structures are projected to separate regions of the joint low dimensional space, which hints to a different organization of the local neighborhoods in the full dimensions (see also Figure S2, Table S1 for a quantative analysis of the agreement of local neighborhoods in the full dimension using SeqAgree/StrAgree measures).

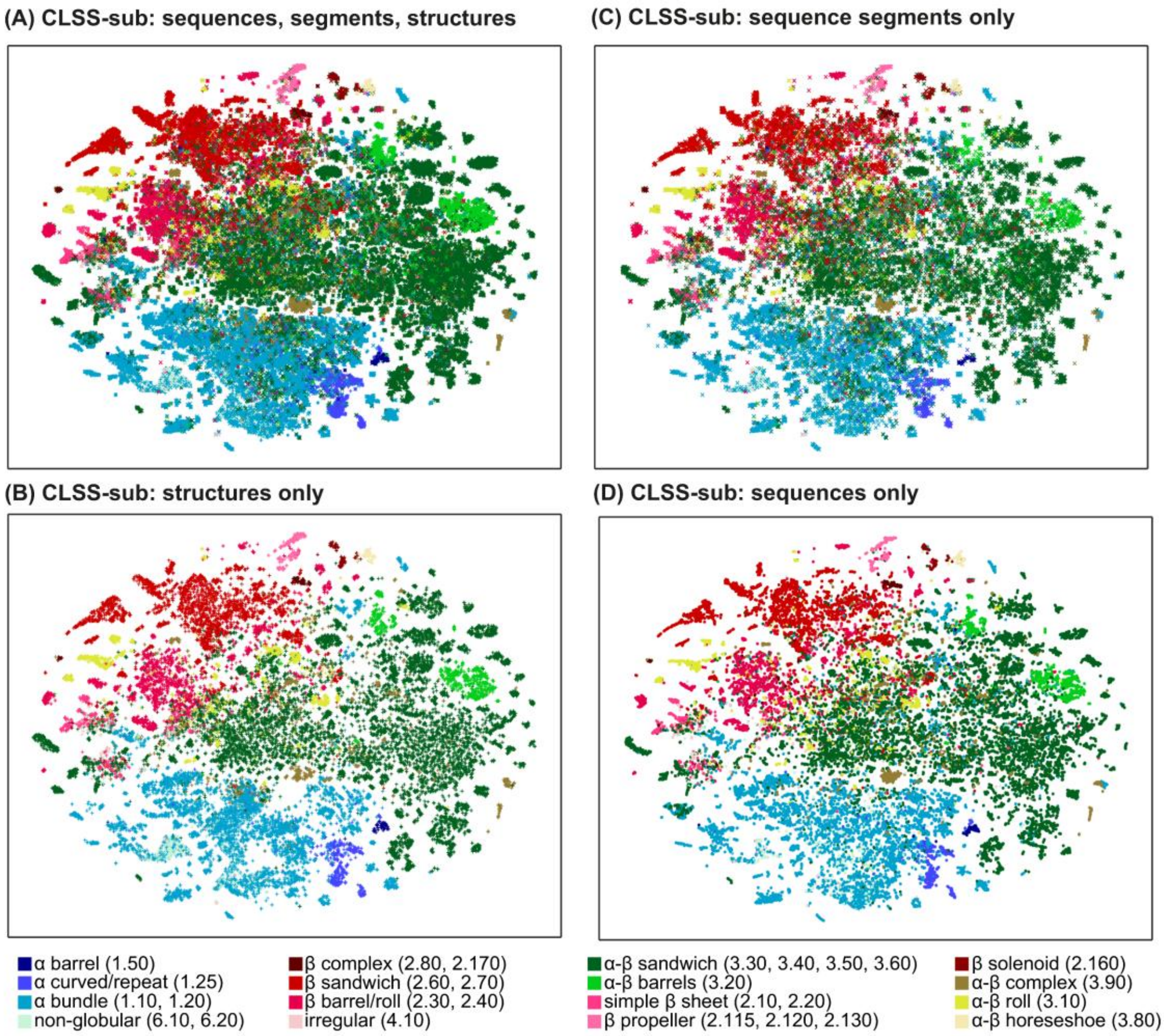

Figure S10: CLSS-sub embedding maps of CATH domains (Dataset 2) – a larger version of Figure S1, first row. For each domain, we calculate the embeddings by three modalities – structure, sequence, and a random sequence segment – and then compute a t-SNE projection of the dataset of all embeddings. Each point represents one of the modalities of a domain colored according to the label of its ECOD architecture. (A) Map of all three modalities. Sequences are marked by circles, structures by '+', and random sequence segments by 'x'. For clarity we show only (B) structure embeddings, (C) sequence segment embeddings, (D) sequence embeddings. We find that the maps of all three modalities are very similar to each other, with the sequence (D) and structure (B) embeddings being the closest. The global organization of the CLSS-sub embedding space positions domains with the same ECOD architecture, and even the same structure class, near each other.

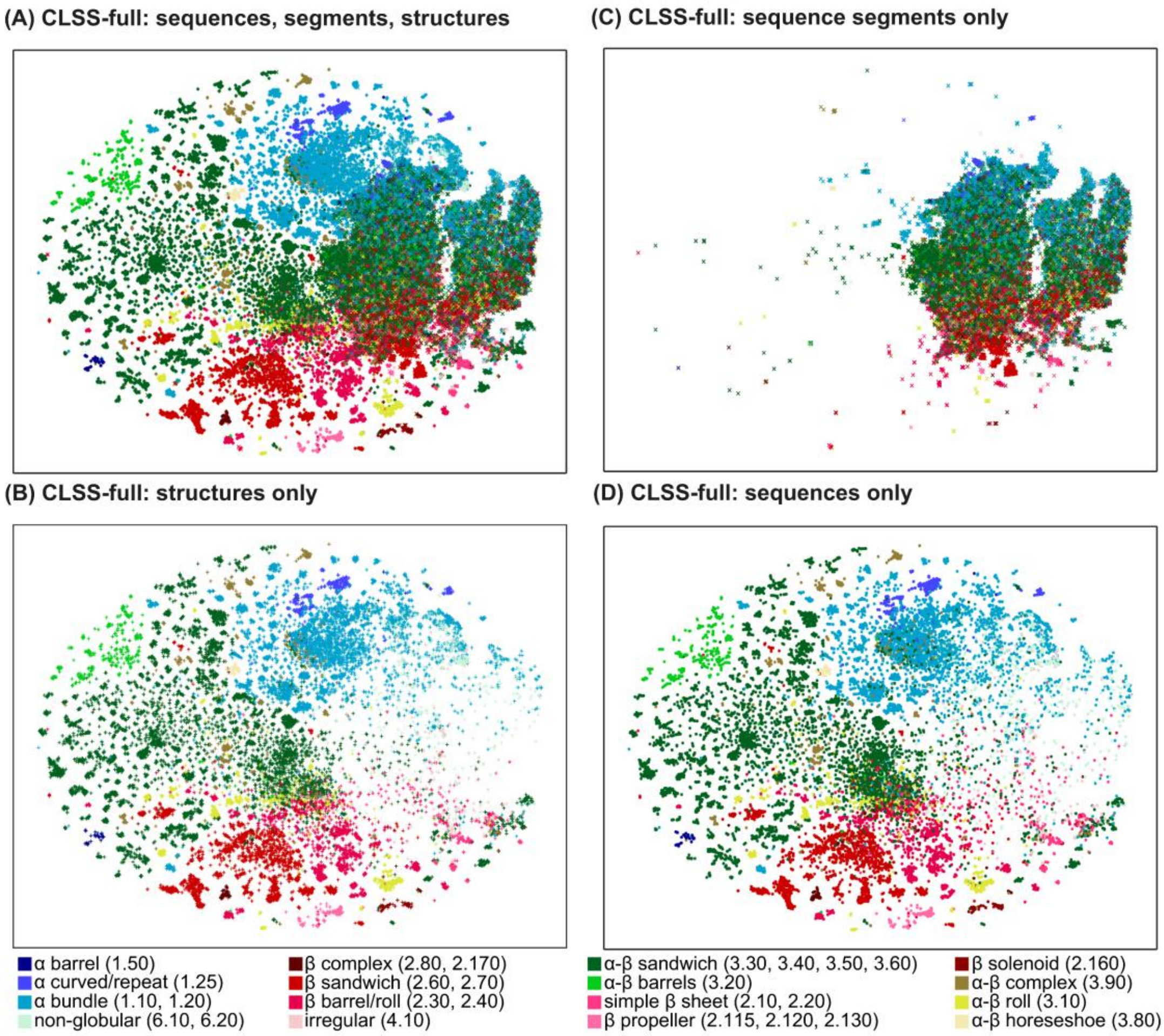

Figure S11: CLSS-full embedding maps of CATH domains (Dataset 2) – a larger version of Figure S1, second row. For each domain, we calculate the embeddings by three modalities – structure, sequence, and a random sequence segment – and then compute a t-SNE projection of the dataset of all embeddings. Each point represents one of the modalities of a domain colored according to the label of its ECOD architecture. (A) Map of all three modalities. Sequences are marked by circles, structures by '+', and random sequence segments by 'x'. For clarity we show only (B) structure embeddings, (C) sequence segment embeddings, (D) sequence embeddings. We find that the maps of all three modalities are very similar to each other, with the sequence (D) and structure (B) embeddings being the closest. CLSS-full maps the subsequence modality to a separate region of the latent space from that of the full sequence and structure modalities, and this modality is far less organized compar:ed to the full sequence and structure modalities. For the full sequence and structure modalities, their maps are generally similar to each other, and similarly position domains with the same CATH architecture, and even the same structure class, near each other

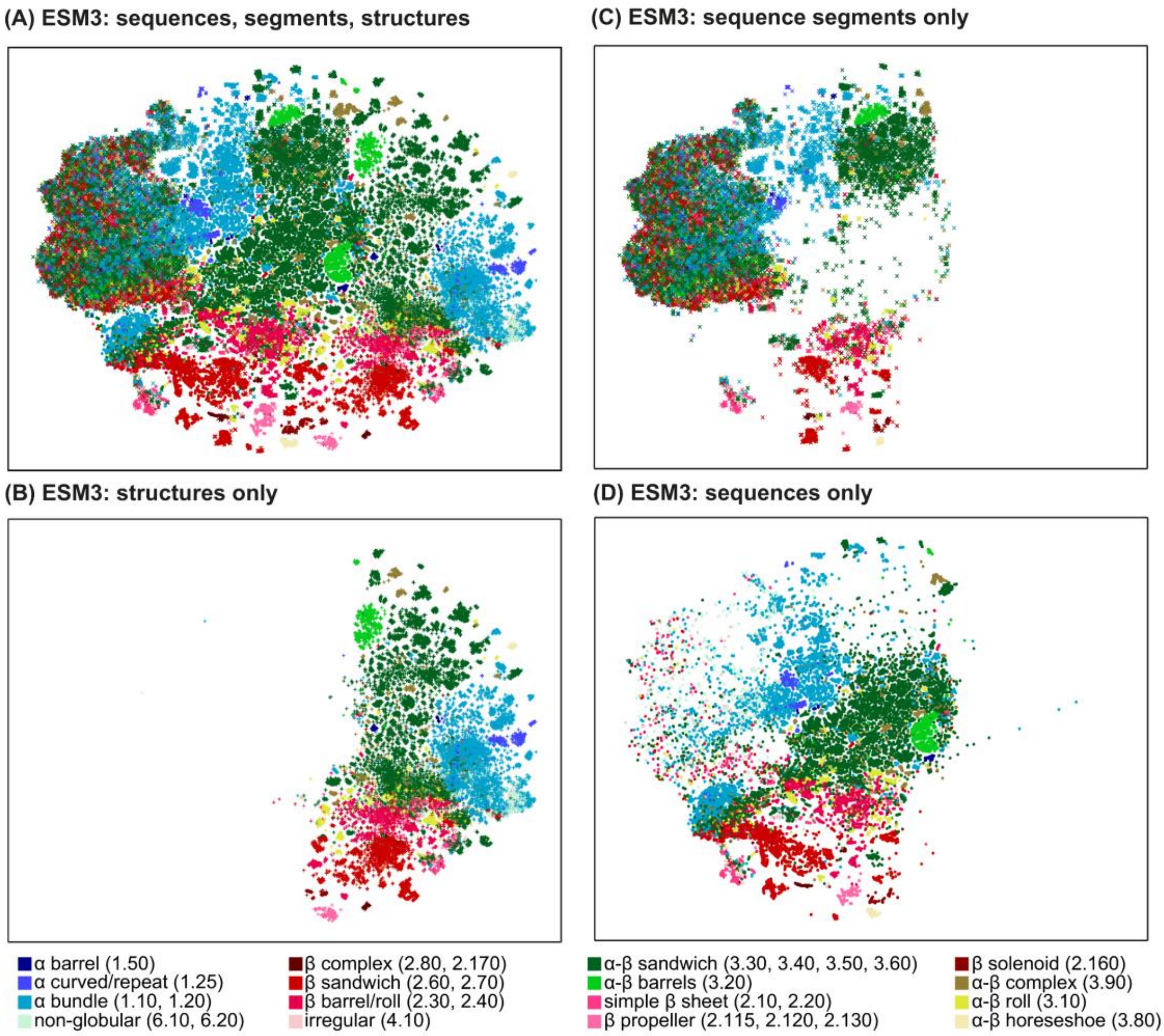

Figure S12: ESM3 maps of CATH domains (Dataset 2) – a larger version of Figure S1, third row. For each domain, we calculate the ESM3 embeddings by the structure, sequence, and a random sequence segment. Each point represents one of the modalities of a domain colored according to the label of its CATH architecture. In this enlarged view we see that the structure modality is best aligned with the CATH labels, the sequence modality less so, and the signal of the embeddings of the random sequence segment is very weak.

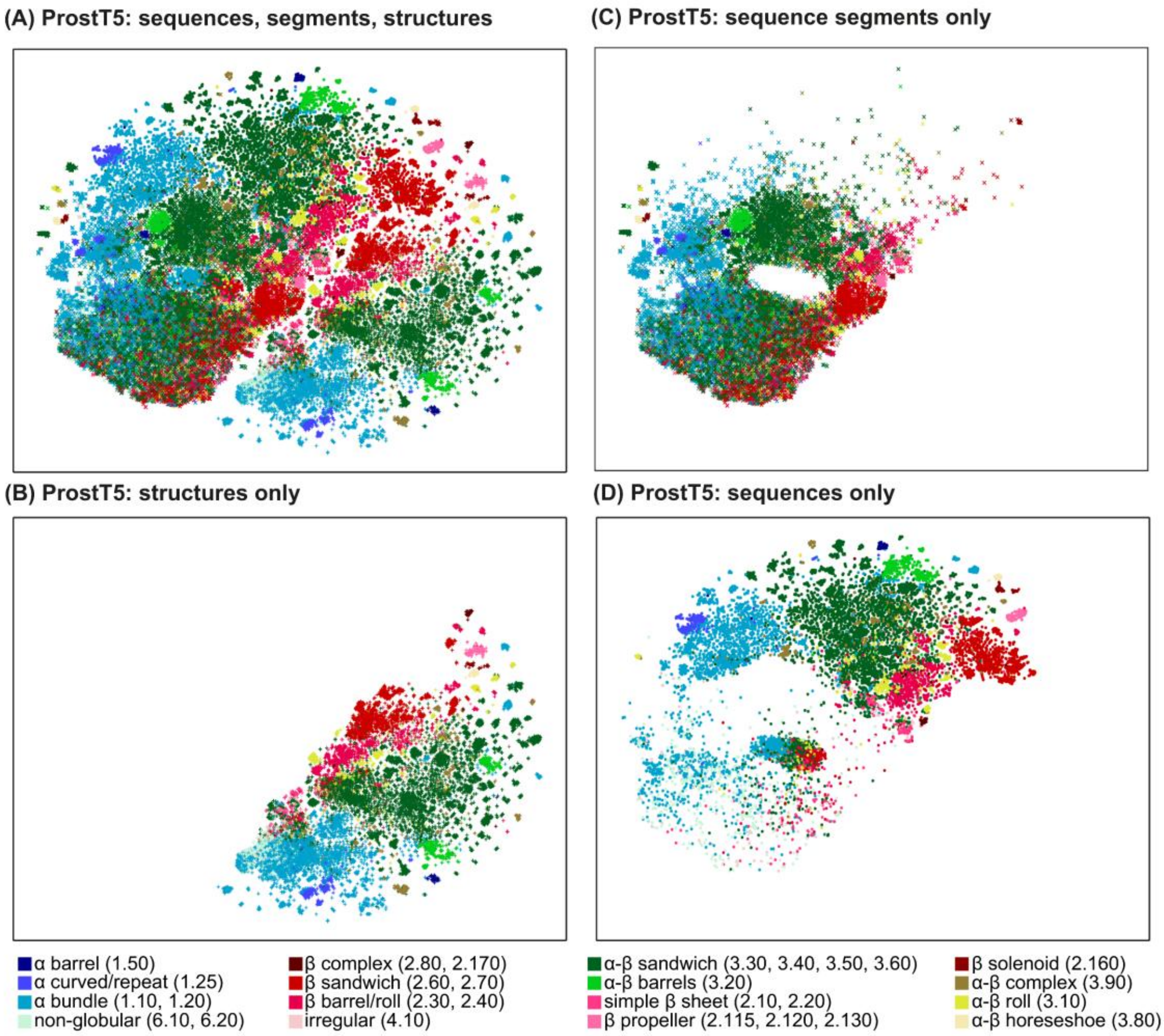

Figure S13: ProstT5 maps of CATH domains (Dataset 2) – a larger version of Figure S1, fourth row. For each domain, we calculate the ProstT5 embeddings by the structure, sequence, and a random sequence segment. Each point represents one of the modalities of a domain colored according to the label of its CATH architecture. In this enlarged view we see that both the structure modality and sequence modality capture the CATH labels well, and the subsequence modality far less so.

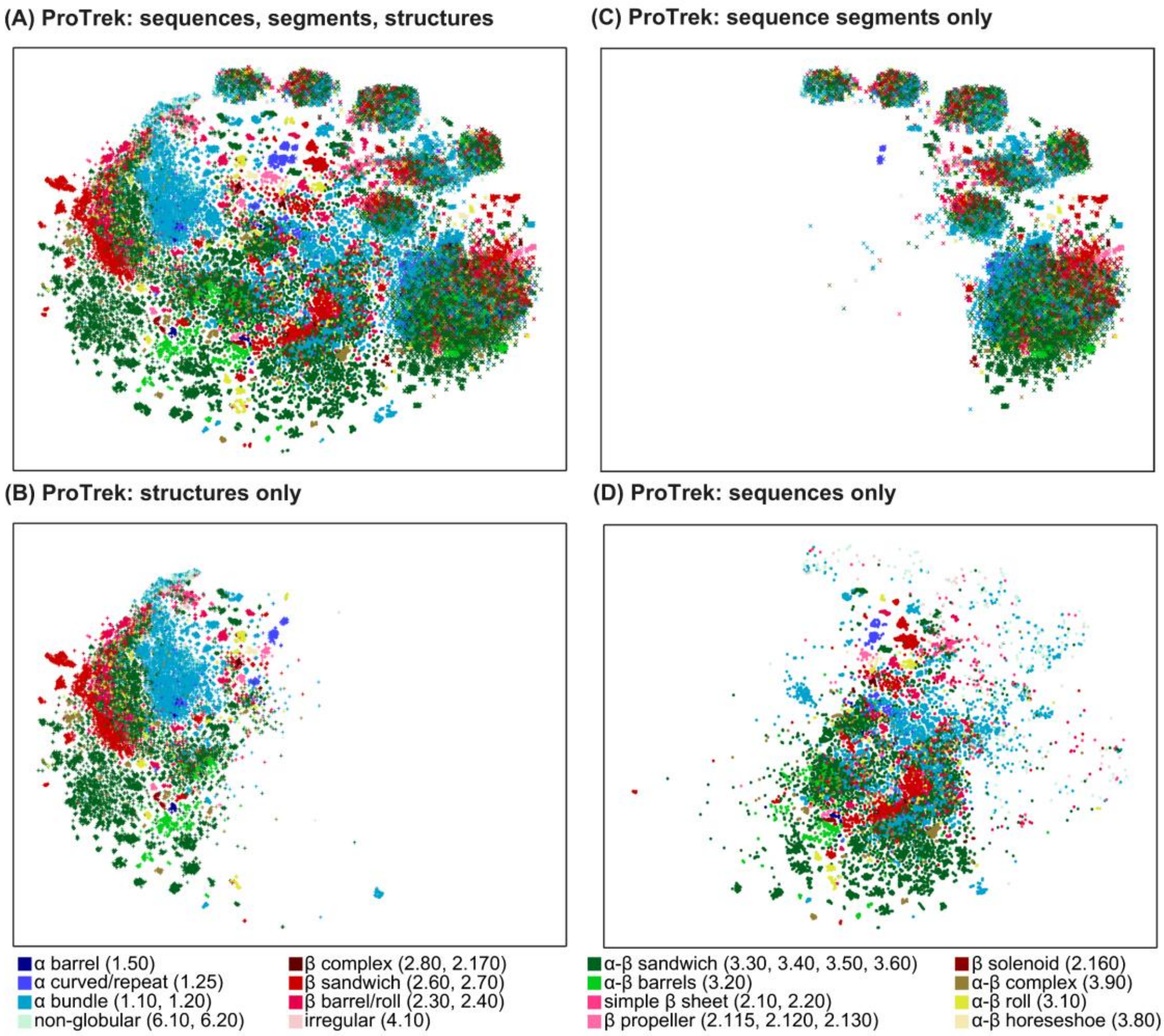

Figure S14: ProTrek maps of CATH domains (Dataset 2) – a larger version of Figure S1, fifth row. For each domain, we calculate the ProTrek embeddings by the structure, sequence, and a random sequence segment. Each point represents one of the modalities of a domain colored according to the label of its CATH architecture. In this enlarged view we see that the agreement of the structure modality with the CATH labels is the strongest, far less so for the sequence modality, and when considering the subsequence modality there is virtually no signal. Also, the embeddings of the sequences and the structures are projected to separate regions of the joint low dimensional space, which hints at a different organization of the local neighborhoods in the full dimensions (see also Figure S2, Table S1 for a quantitative analysis of the agreement of local neighborhoods in the full dimension using SeqAgree/StrAgree measures).

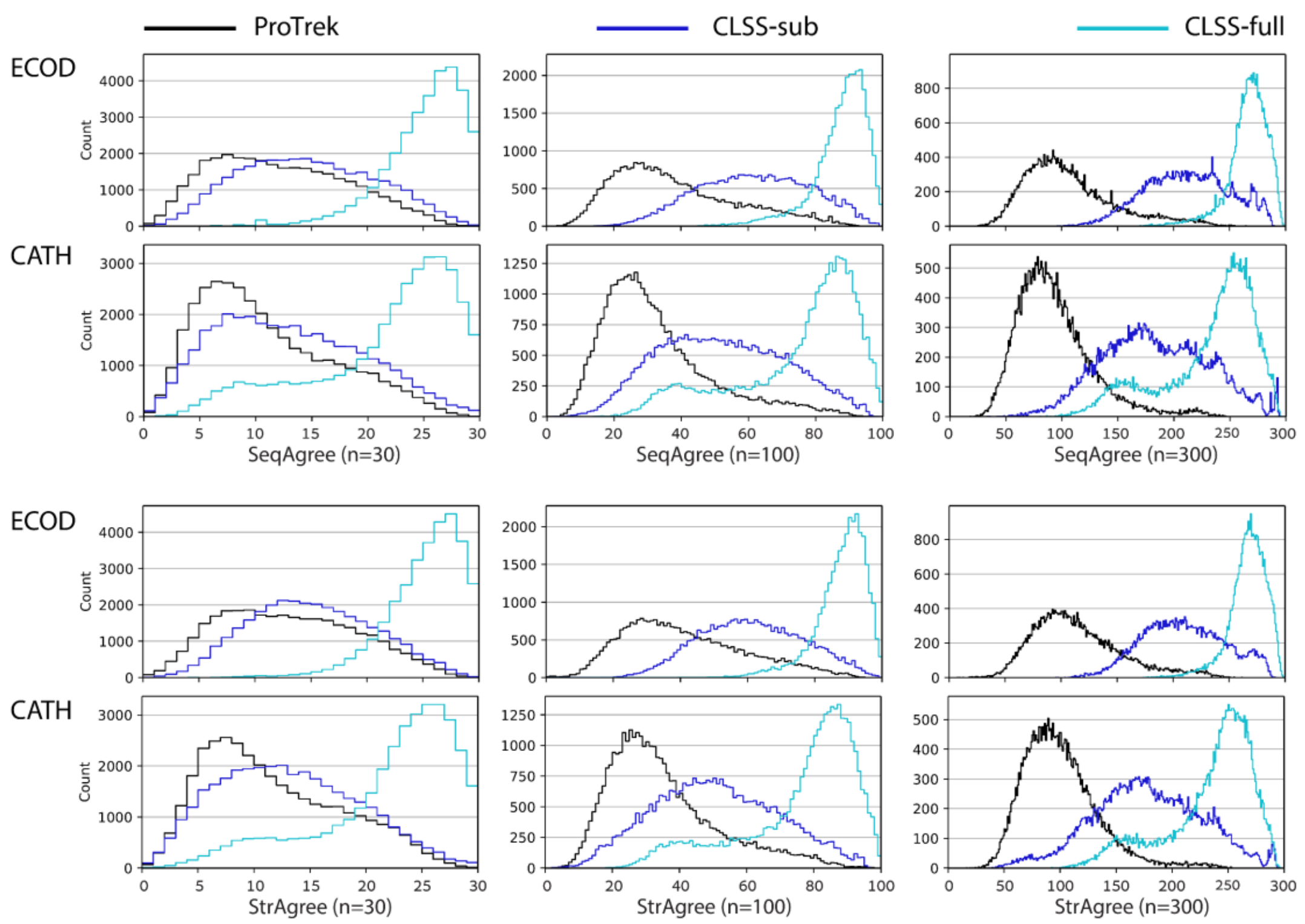

Figure S15: Distributions of SeqAgree (top two rows) and StrAgree (bottom two rows) values for neighborhoods of size 30 (left), 100 (middle), and 300 (right). In each case we consider both Datasets 1 (ECOD), and 2 (CATH), and compare three models: CLSS-sub, CLSS-full, and ProTrek. A model that embeds the sequence and structure modality more consistently, will have distributions that are shifted to the right (higher values). We see that the top performer in all cases is CLSS-full, followed by CLSS-sub, and then ProTrek.

			Number of neighbors						
Measure	DB	Model	10	30	50	100	150	200	300
SeqAgree%	ECOD	CLSS-sub	34.6	47.33	53.93	61.25	64.71	67.3	70.51
	CATH		31.9	41.78	46.32	51.75	54.99	57.42	60.87
	ECOD	CLSS-full	75.6	81.76	84.3	87.12	87.88	88.37	88.68
	CATH		64.66	70	72.16	74.43	75.49	76.24	77.04
	ECOD	ProTrek	39.13	39.12	38.89	37.69	36.65	36	35.42
	CATH		36.56	34.88	33.74	32.05	31.25	30.93	30.94
StrAgree%	ECOD	CLSS-sub	38.65	48.71	54.22	60.76	64.07	66.55	69.66
	CATH		34.45	42.05	45.68	50.41	53.35	55.67	59.01
	ECOD	CLSS-full	76.58	82.2	84.71	87.36	88.1	88.56	88.82
	CATH		66.29	71.39	73.47	75.56	76.53	77.18	77.86
	ECOD	ProTrek	41.01	41.96	41.99	40.75	39.57	38.75	37.91
	CATH		38.44	37.45	36.37	34.69	33.74	33.32	33.19

Table S1: Mean SeqAgree/ StrAgree consistency measure values (in %) for the models CLSS-sub, CLSS-full, and ProTrek, for Datasets 1 (ECOD), and 2 (CATH). The neighborhood sizes vary from 10 to 300.

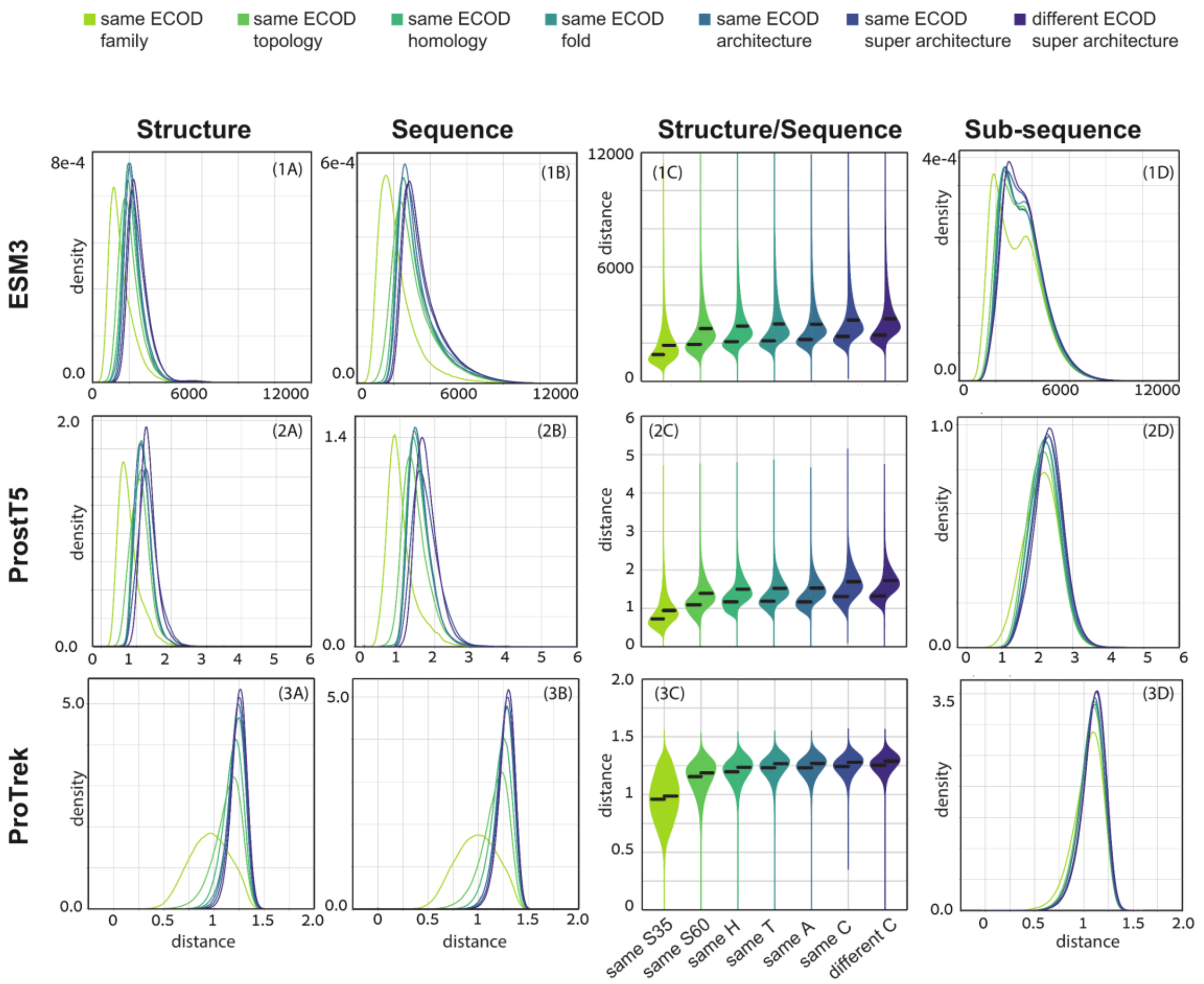

Figure S16: Comparison to Figure 3 for the ESM3, ProstT5, and ProTrek Models. The estimated distributions of distances between pairs of embeddings for domains in Dataset 1 by these models. The distributions of distances of domain pairs with the same ECOD label at different levels of the hierarchy. The color palette indicates the shared level in the ECOD hierarchy, from pairs with the same H-group label in light-green through pairs with different structure class in purple. The leftmost column shows the distances for the structure modality, then the full sequence modality, and the subsequence modality is in the rightmost column. The violin plots pair the distributions of distances of the full sequence and structure modalities. The distributions of ESM3 and ProstT5 differ between the full sequence and structure modalities, and are most similar for ProTrek. The distributions for the subsequence modality are all the same, regardless of the level of similarity in the ECOD hierarchy.

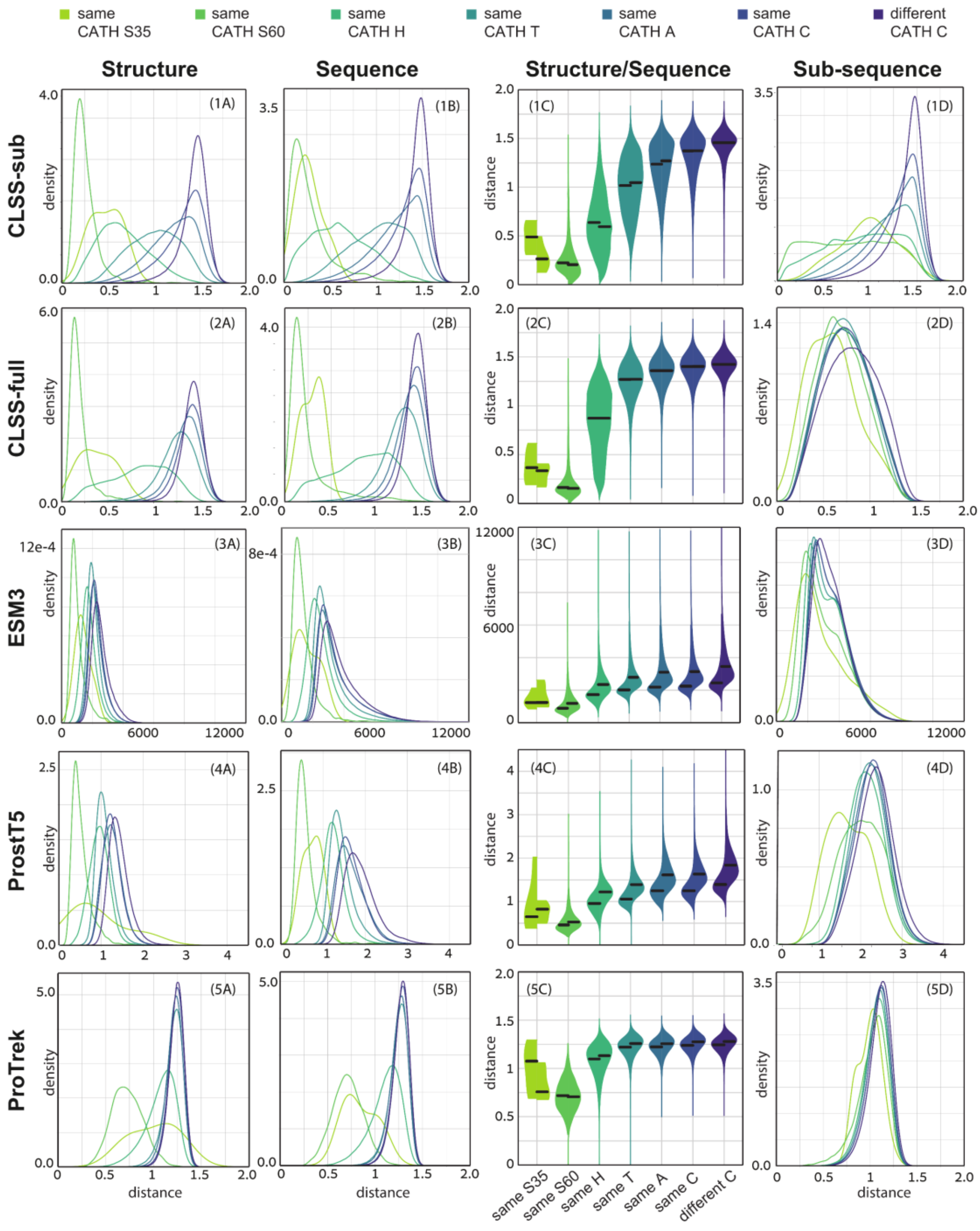

Figure S17: The estimated distributions of distances between pairs of embeddings for domains in Dataset 2 by CLSS and compared models. The distributions of distances of domain pairs with the same CATH label at different levels of the hierarchy. The color palette indicates the shared level in the CATH hierarchy, from pairs with the same S35-group label in light-green through pairs with different 'C' label in purple. The leftmost column shows the distances for the structure modality, then the full sequence modality, and the subsequence modality is in the rightmost column. The violin plots pair the distributions of distances of the full sequence and structure modalities. Similarly to the plots for the ECOD dataset (Figures 3, S13), only CLSS-sub models the sub-sequence modality well. The CLSS models and ProTrek have symmetric distributions for the structure and full sequence modalities.

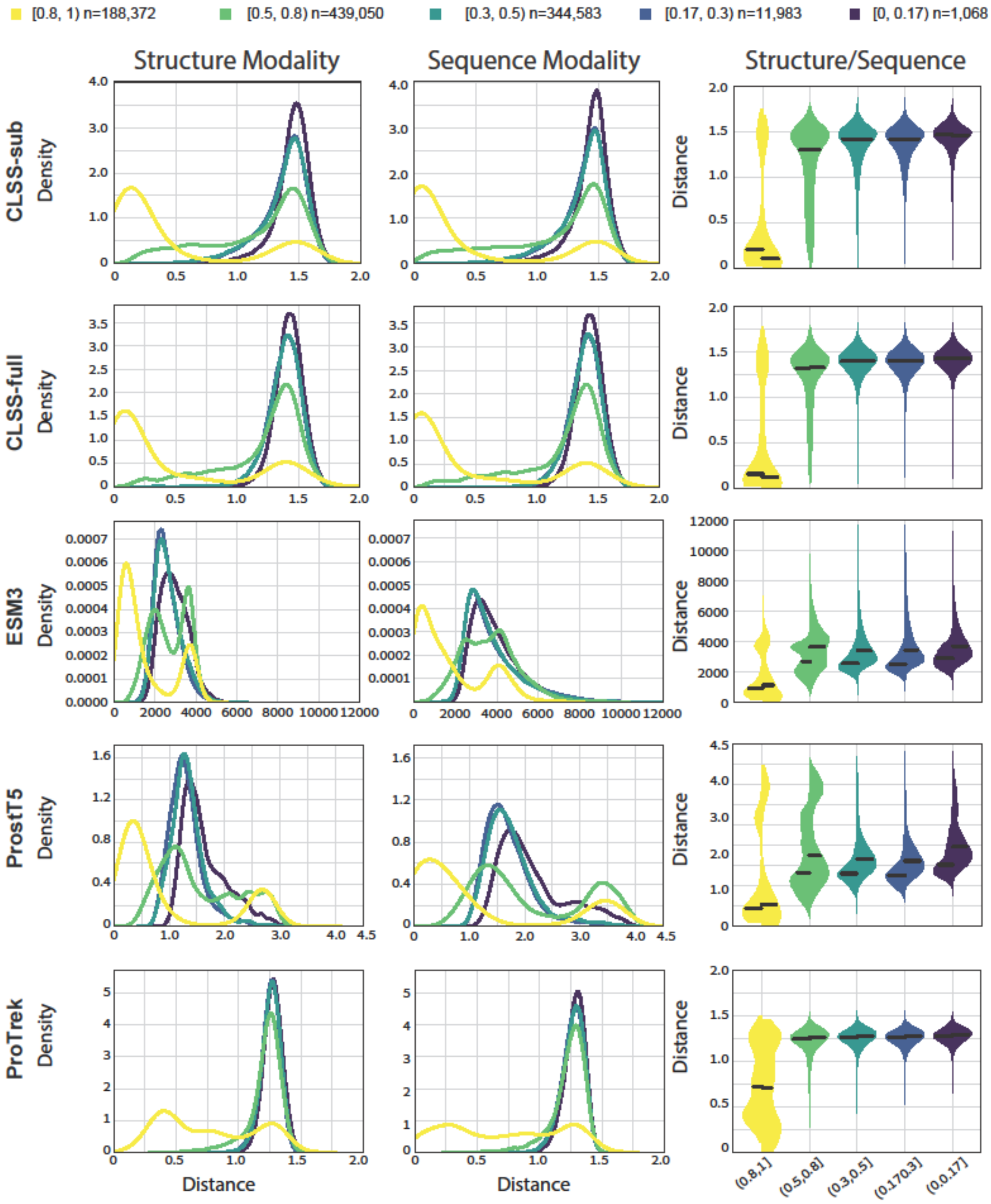

Figure S18: The estimated distributions of distances between pairs of embeddings for 933 domains with pre-calculated TM-align scores in the ProteinShake protein similarity dataset, grouped by their TM-align scores. We calculate the embedding distances in the structure and sequence modalities, using CLSS-sub, CLSS-full, and the PLMs ESM3, ProstT5, and ProTrek. The sets of pairs are ones with TM-scores that range from very good (TM-score > 0.8) in yellow, via good (0.8 > TM-score > 0.5), in light green, to even less good (the ranges are listed on top). We see that for all models, only high TM-score pairs (yellow) are consistently embedded near each other. The violin plots in the 3rd column show that the embedding distance distributions from the CLSS models are similar across both the sequence and structure modalities, and this is not the case for ESM3 and ProstT5.

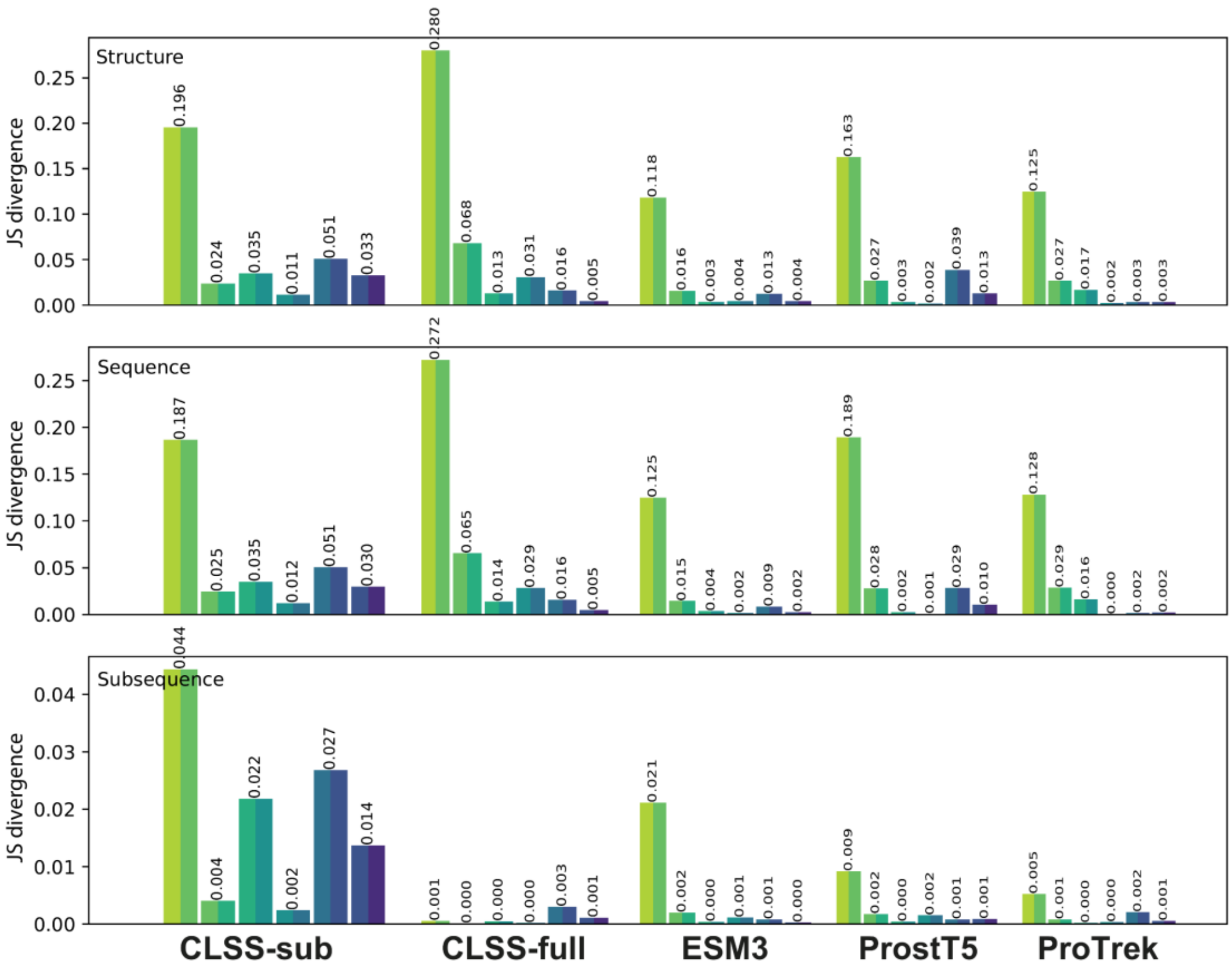

Figure S19: The Jensen-Shannon divergence values for neighboring distributions in Dataset 1 with ECOD labels (Figure S13). For each combination of model and modality, we consider the 6 combinations of neighboring distributions (e.g., same ECOD family vs. same ECOD topology, shown with a bar of yellow and light green). Higher values of Jensen-Shannon divergence suggest that the distributions are more distinct from one another. We see that the greatest separations for the structure and full sequence modalities (first and second rows) are for the CLSS-full model. Also, for the subsequence modality (third row), only CLSS-sub meaningfully separates all levels of the hierarchy.

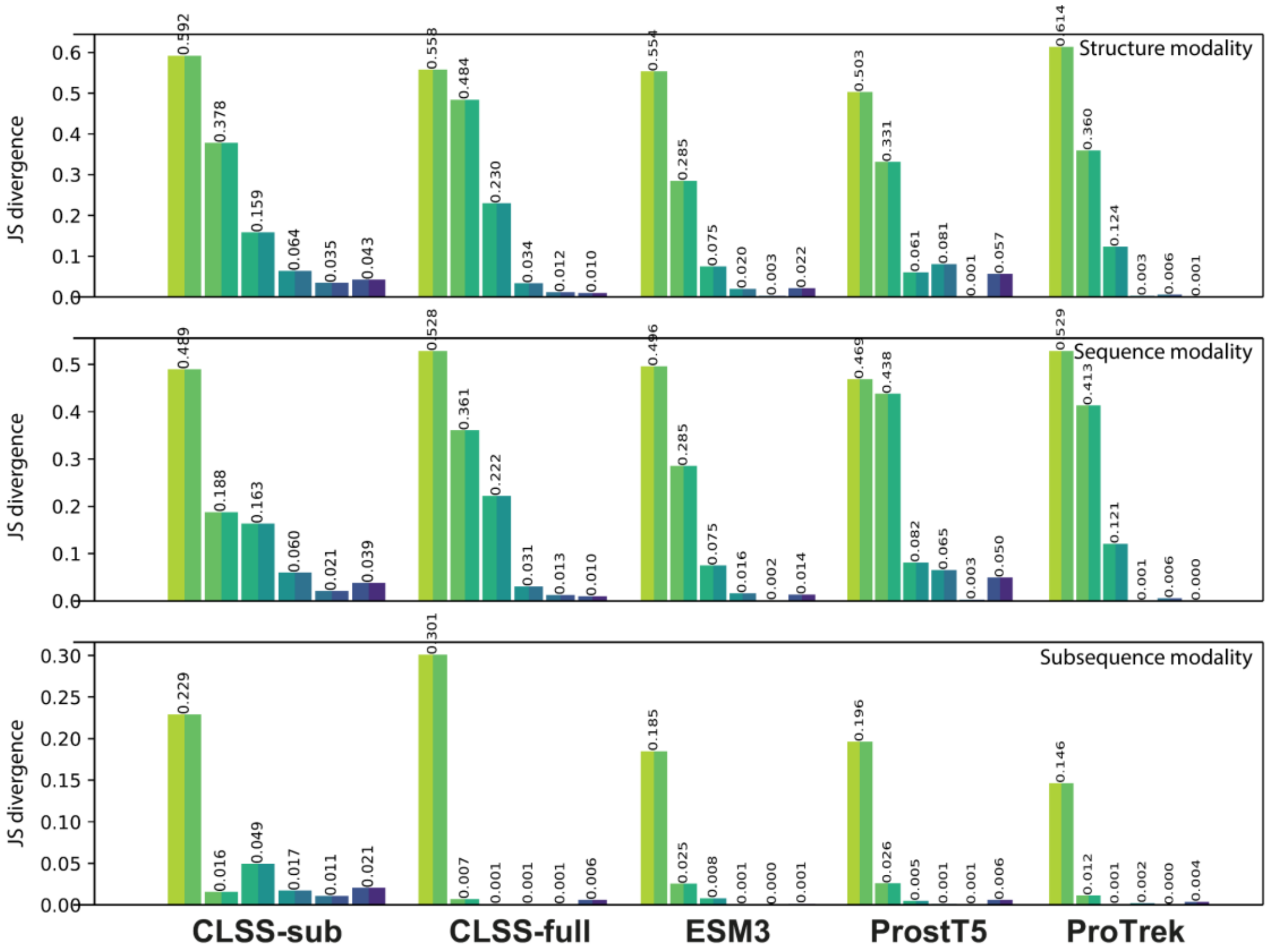

Figure S20: The Jensen-Shannon divergence values for neighboring distributions in Dataset 2 with CATH labels (Figure S15). For each combination of model and modality, we consider the 6 combinations of neighboring distributions (e.g., same CATH S35 vs. same CATH S60, shown with a bar of yellow and light green). Higher values of Jensen-Shannon divergence suggest that the distributions are more distinct from one another. We see that the greatest separations for the structure and full sequence modalities (first and second rows) are for the CLSS-full model and ProstT5. Also, for the subsequence modality (third row), CLSS-sub is the model that most meaningfully separates all levels of the hierarchy.

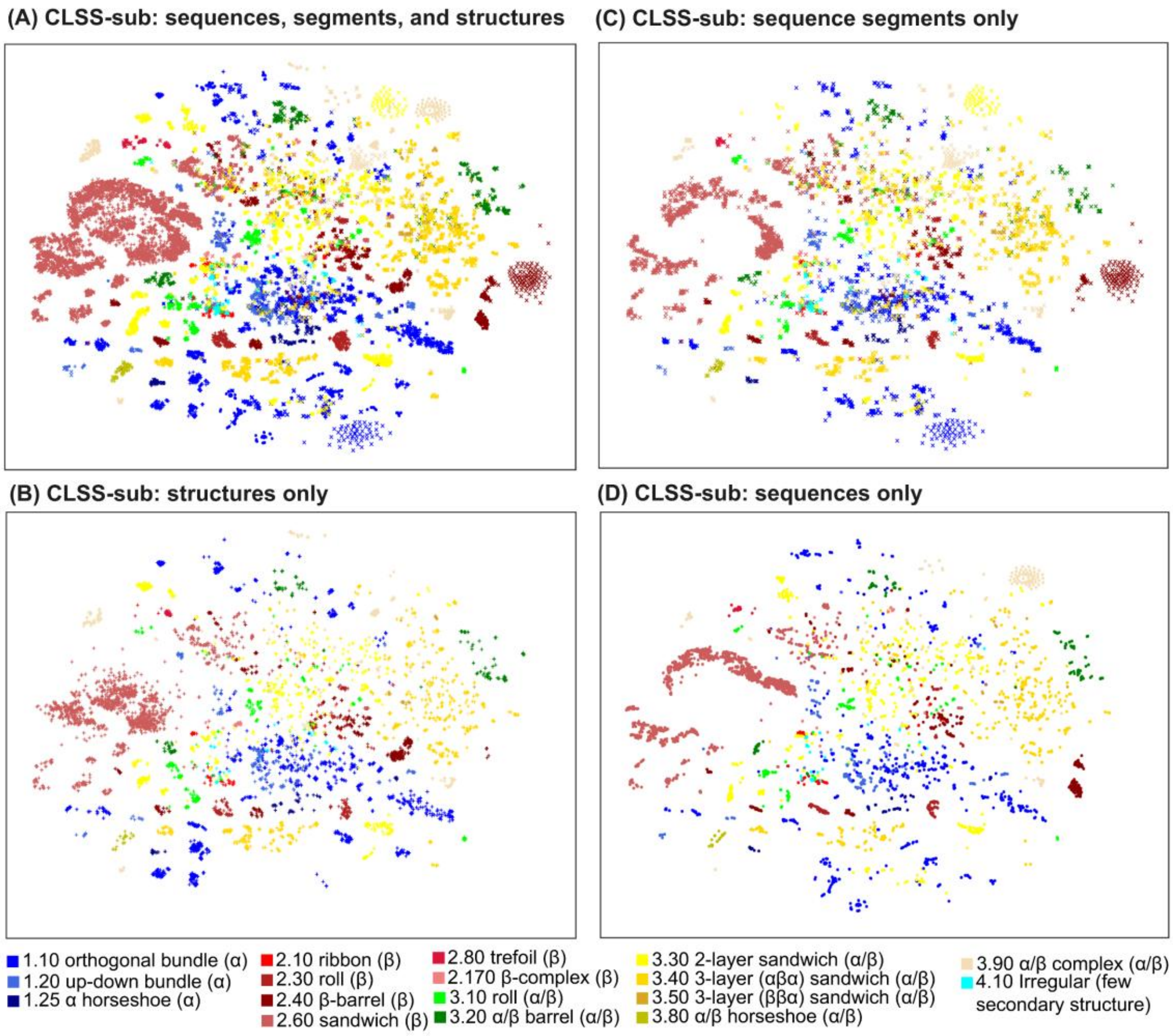

Figure S21: Maps derived from CLSS-sub embeddings of CATH domains with similar sequences and topologically different structures (Dataset 3). For each domain, we consider three modalities: sequence, structure, and a random sequence segment, and compute a t-SNE projection of the embeddings. Each point represents one of the modalities of one domain colored according to the label of its CATH architecture. (A) All three modalities, and only (B) structure embeddings, (C) sub-sequence embeddings, and (D) sequence embeddings. Even for this challenging dataset, the three modalities produce overall similar maps.

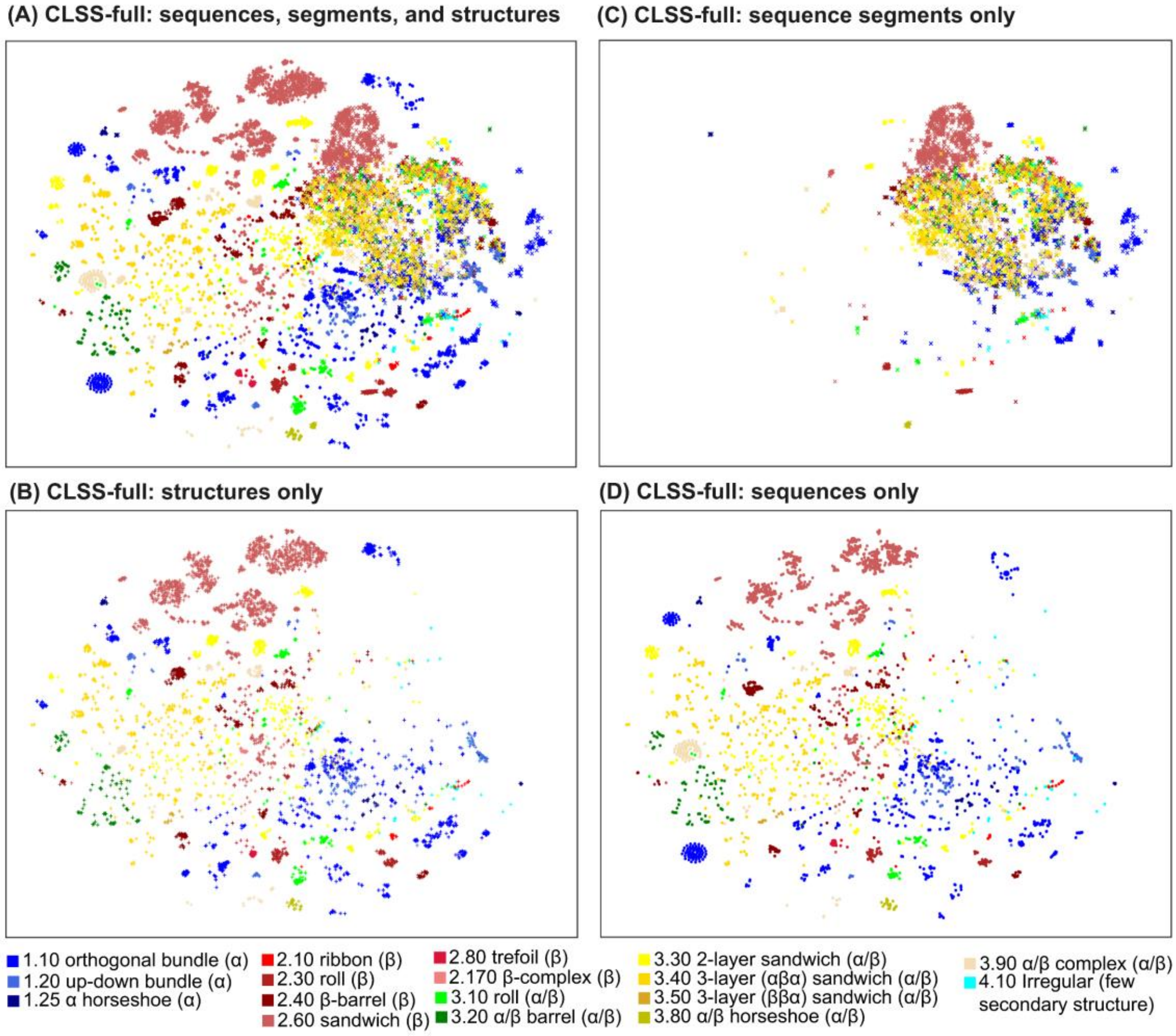

Figure S22: Maps derived from CLSS-full embeddings of CATH domains with similar sequences and topologically different structures (Dataset 3). For each domain, we consider three modalities: sequence, structure, and a random sequence segment, and compute a t-SNE projection of the embeddings. Each point represents one of the modalities of one domain colored according to the label of its CATH architecture. (A) All three modalities, and only (B) structure embeddings, (C) sub-sequence embeddings, and (D) sequence embeddings. Similarly to the maps for Datasets 1 (Figure S4) and 2 (Figure S9), we see that the full sequence and structure modalities are co-embedded, and the maps of these modalities are similar. In contrast, the subsequence modality is not co-embedded by this model next to the full sequence and structure modality, and its signal appears non-informative.

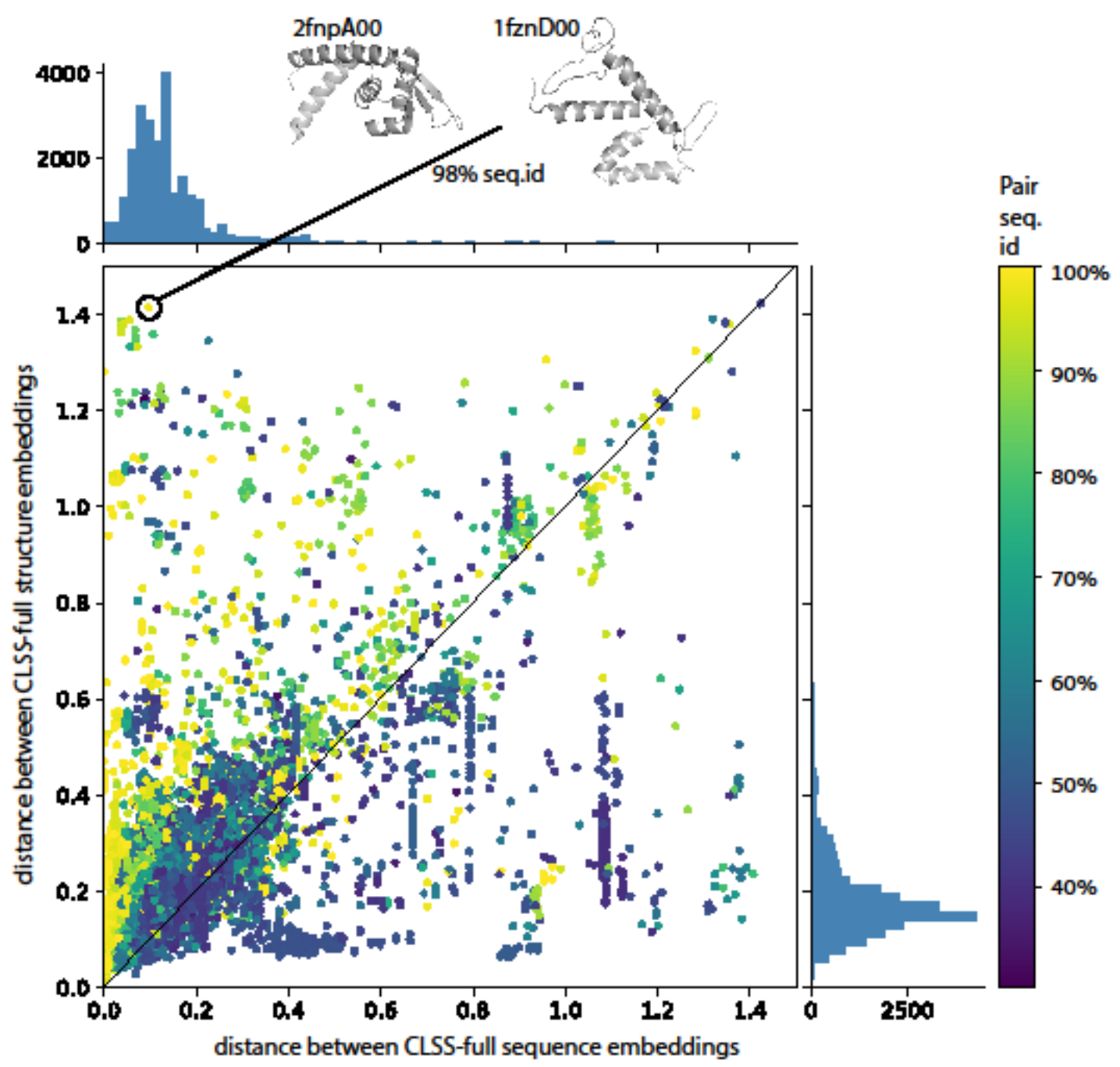

Figure S23: The distance between the embeddings of the sequence modality vs. the distance between the embeddings of the structure modality calculated using the CLSS-full model, for the pairs in [46]. The points are colored according to the % pairwise sequence identity as reported in [46]. Along the x, y-axes we show the histograms of these distances. We see that as expected from the design of Dataset 3, the distances among the sequences are smaller than those among the structures. The extreme cases that we looked at behave as expected: for example, the structures of the pair marked are shown: these are 2fnpA00 and 1fznD00 (98% sequence identity, and the structure and sequence based RMSDs are 8.4A, and 17.2A respectively).

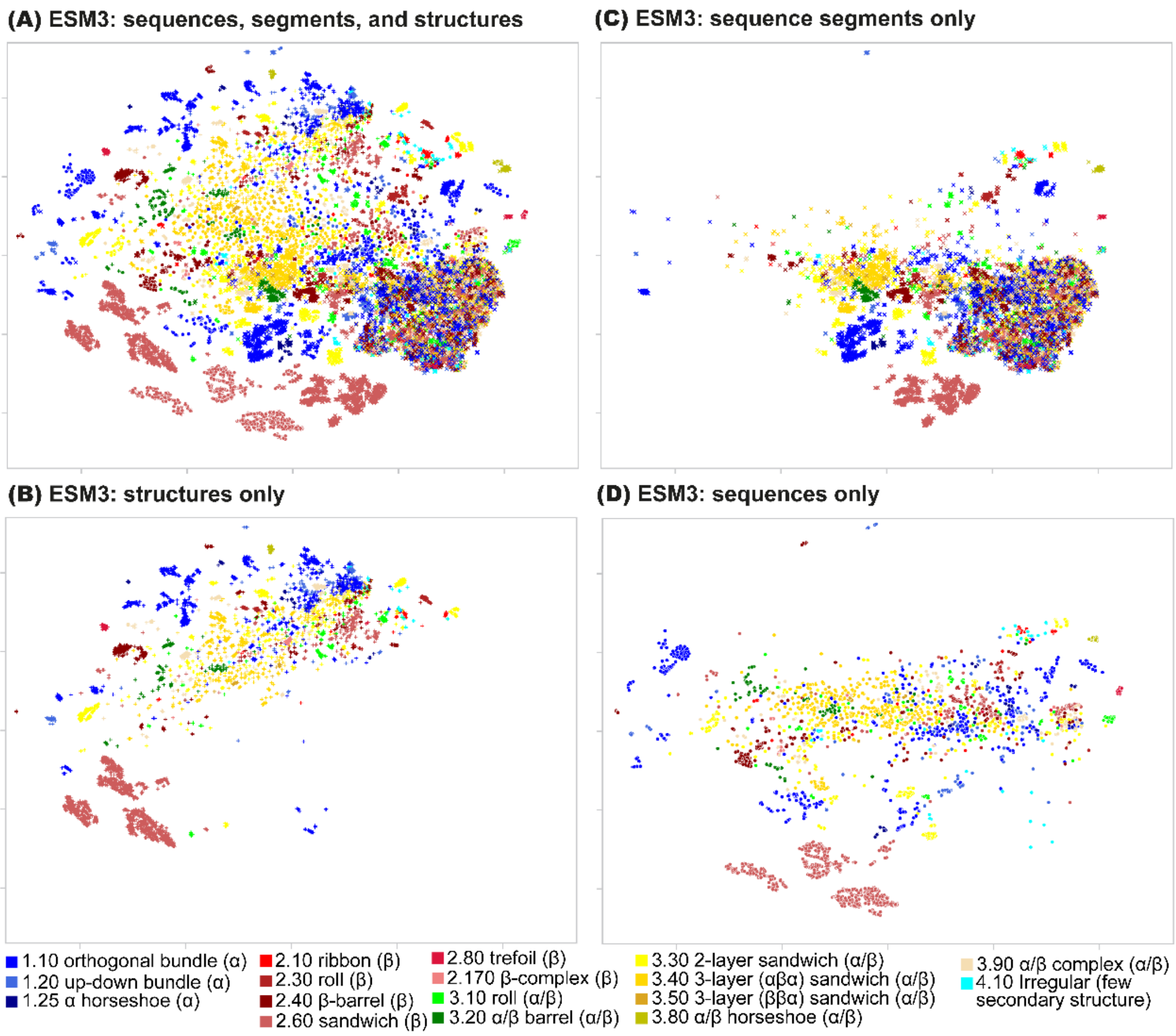

Figure S24: Maps derived from ESM3 embeddings of CATH domains with similar sequences and topologically different structures (Dataset 3). For each domain, we consider three modalities: sequence, structure, and a random sequence segment, and compute a t-SNE projection of the embeddings. Each point represents one of the modalities of one domain colored according to the label of its CATH architecture. (A) All three modalities, and only (B) structure embeddings, (C) sub-sequence embeddings, and (D) sequence embeddings. Even though the sequence and structure modalities are not co-embedded near each other, ESM3 embeddings of these metamorphic proteins fail to successfully group the proteins according to their CATH architecture in both these modalities. In the sequence sub-segment modality, the signal is generally lost.

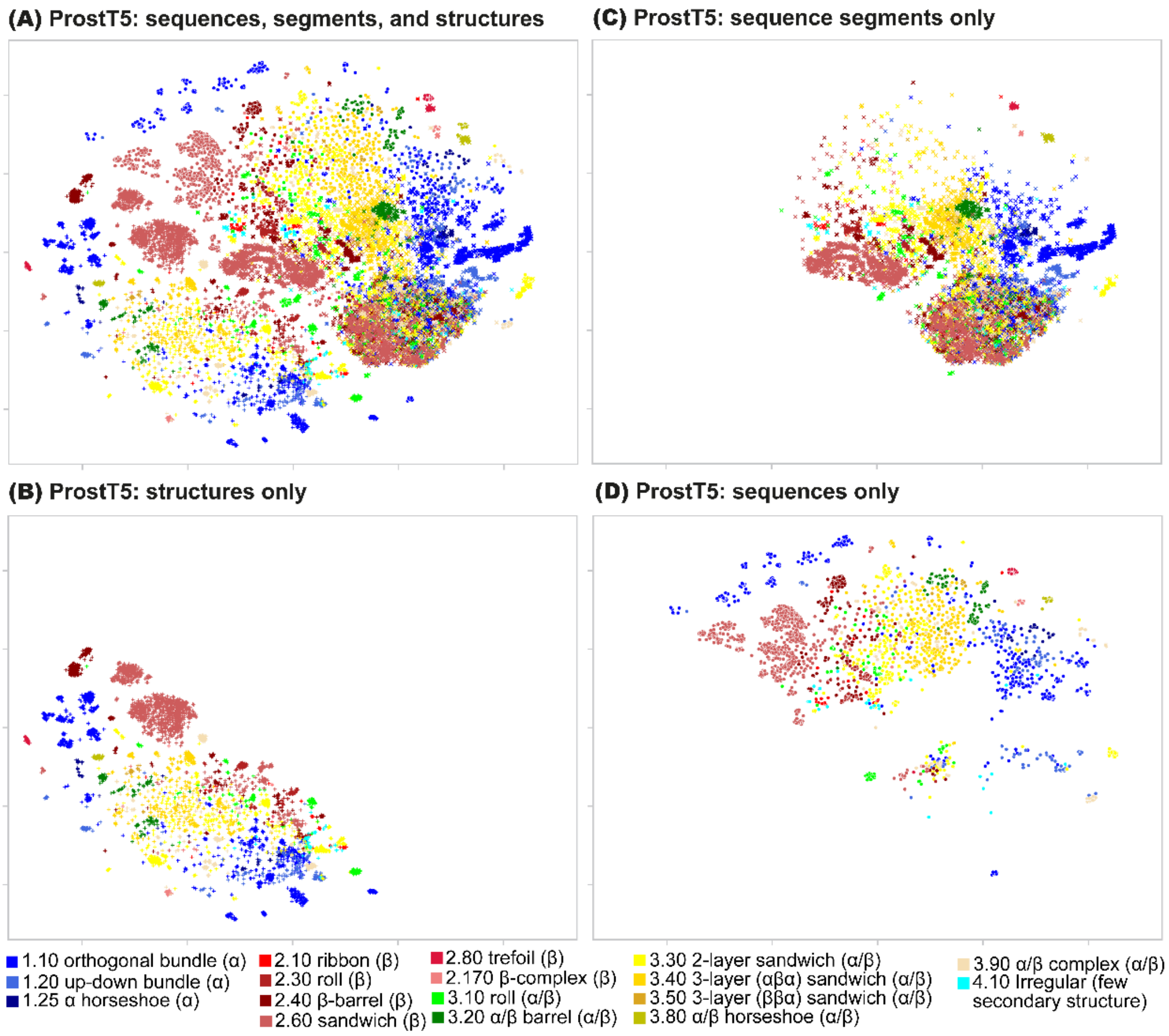

Figure S25: Maps derived from ProstT5 embeddings of CATH domains with similar sequences and topologically different structures (Dataset 3). For each domain, we consider three modalities: sequence, structure, and a random sequence segment, and compute a t-SNE projection of the embeddings. Each point represents one of the modalities of one domain colored according to the label of its CATH architecture. (A) All three modalities, and only (B) structure embeddings, (C) sub-sequence embeddings, and (D) sequence embeddings. ProstT5 successfully groups the proteins according to their CATH architecture in the structure and sequence modalities, but in the sequence sub-segment modality, the signal is generally lost.

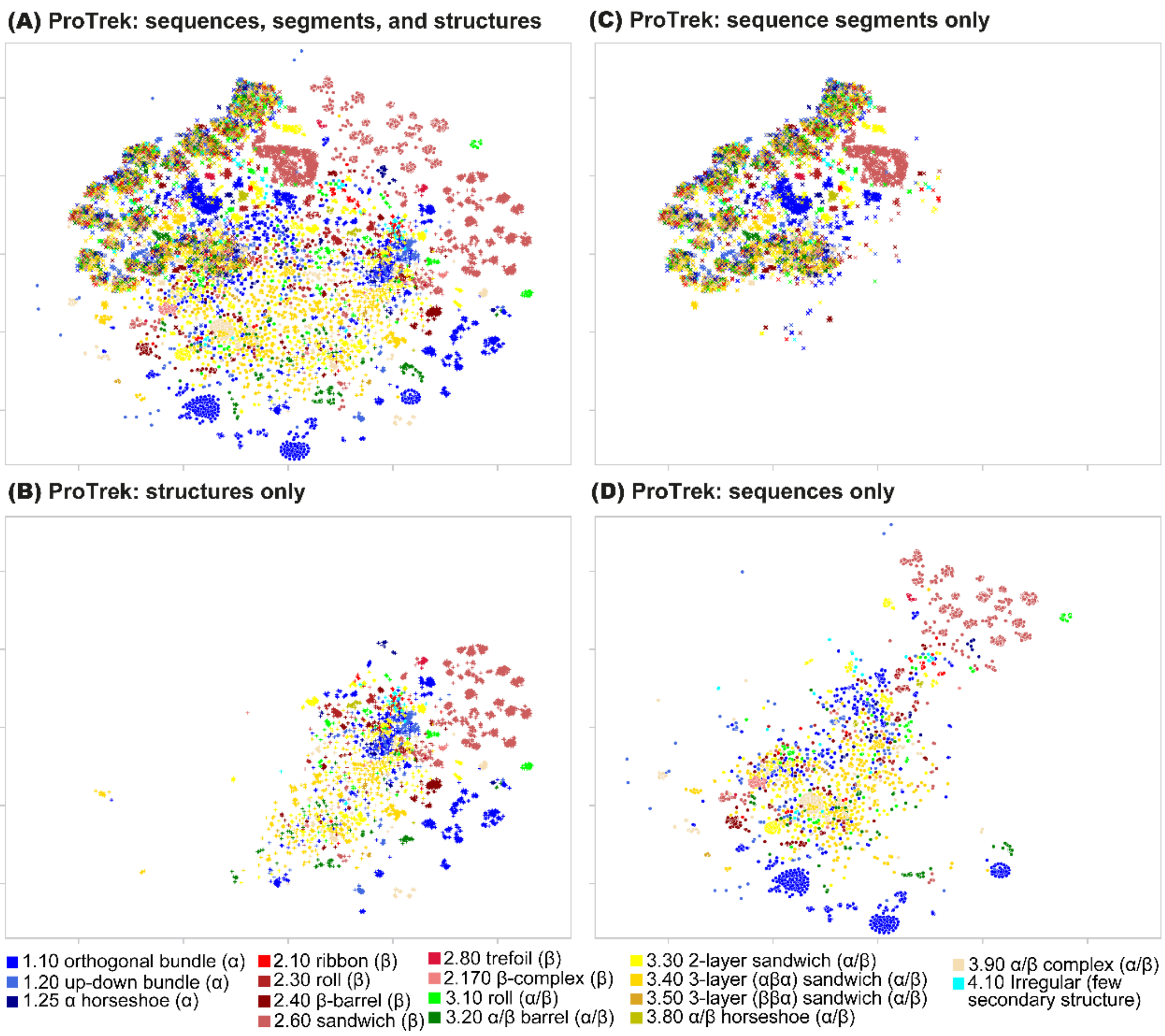

Figure S26: Maps derived from ProTrek embeddings of CATH domains with similar sequences and topologically different structures (Dataset 3). For each domain, we consider three modalities: sequence, structure, and a random sequence segment, and compute a t-SNE projection of the embeddings. Each point represents one of the modalities of one domain colored according to the label of its CATH architecture. (A) All three modalities, and only (B) structure embeddings, (C) sub-sequence embeddings, and (D) sequence embeddings. For this dataset too, the ProTrek embeddings of the full sequences and the structure modalities, do not occupy the same region of the projected space, suggesting their organization in the full dimension is different. Also, in the sub-sequence, the signal is generally lost.

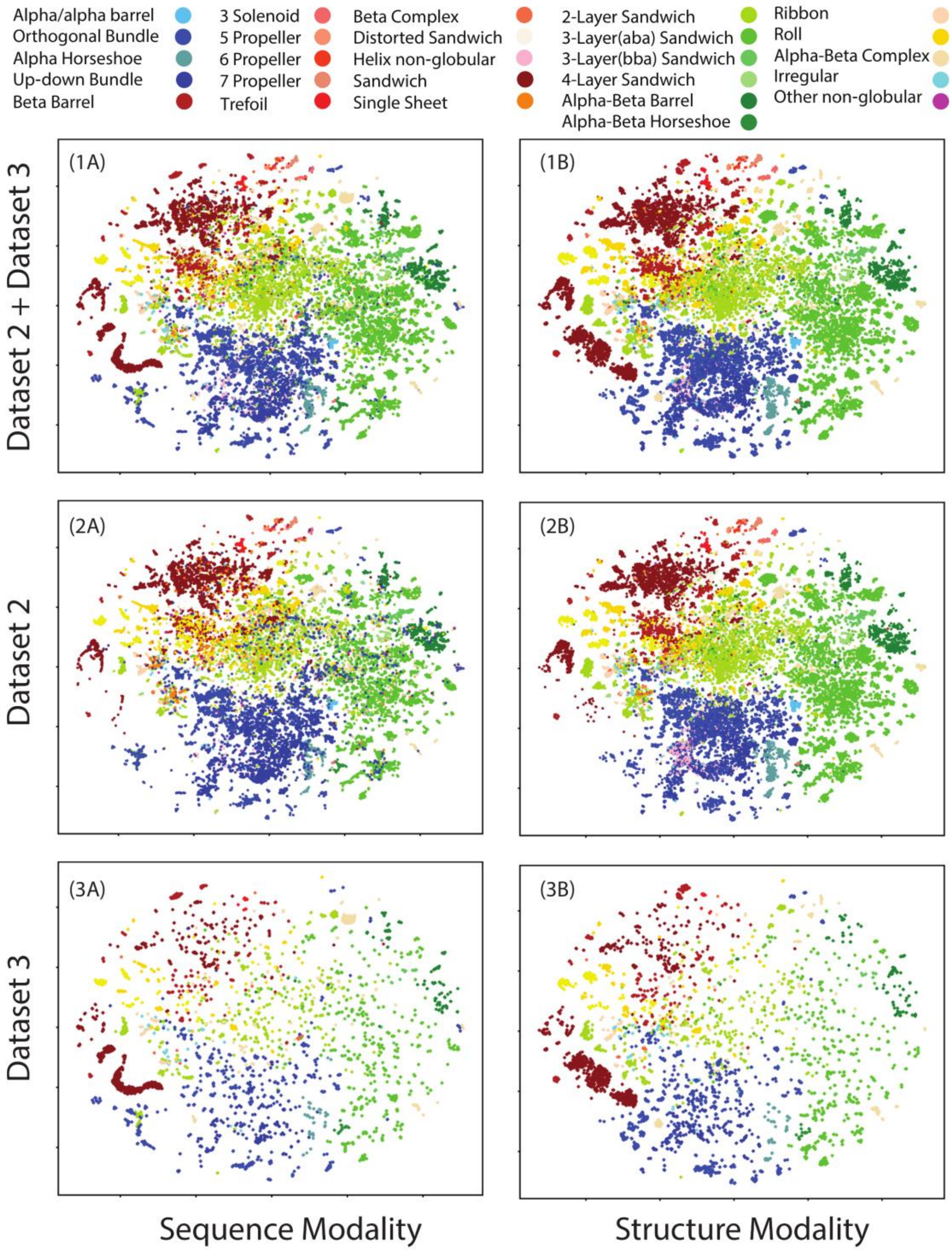

Figure S27: Further characterization of CLSS embeddings of Dataset 3 of metamorphic proteins, when mapped together with the CATH domains in Dataset 2. To place the embeddings of Dataset 3 within the context of protein structure space, we calculated the t-SNE projection of the sequence and structure embeddings of the CATH domains of Dataset 2 and the sequence and structure domains of the metamorphic domains in Dataset 3. The embeddings are colored by architecture. We see that generally, the metamorphic proteins come from across all of CATH domain space.

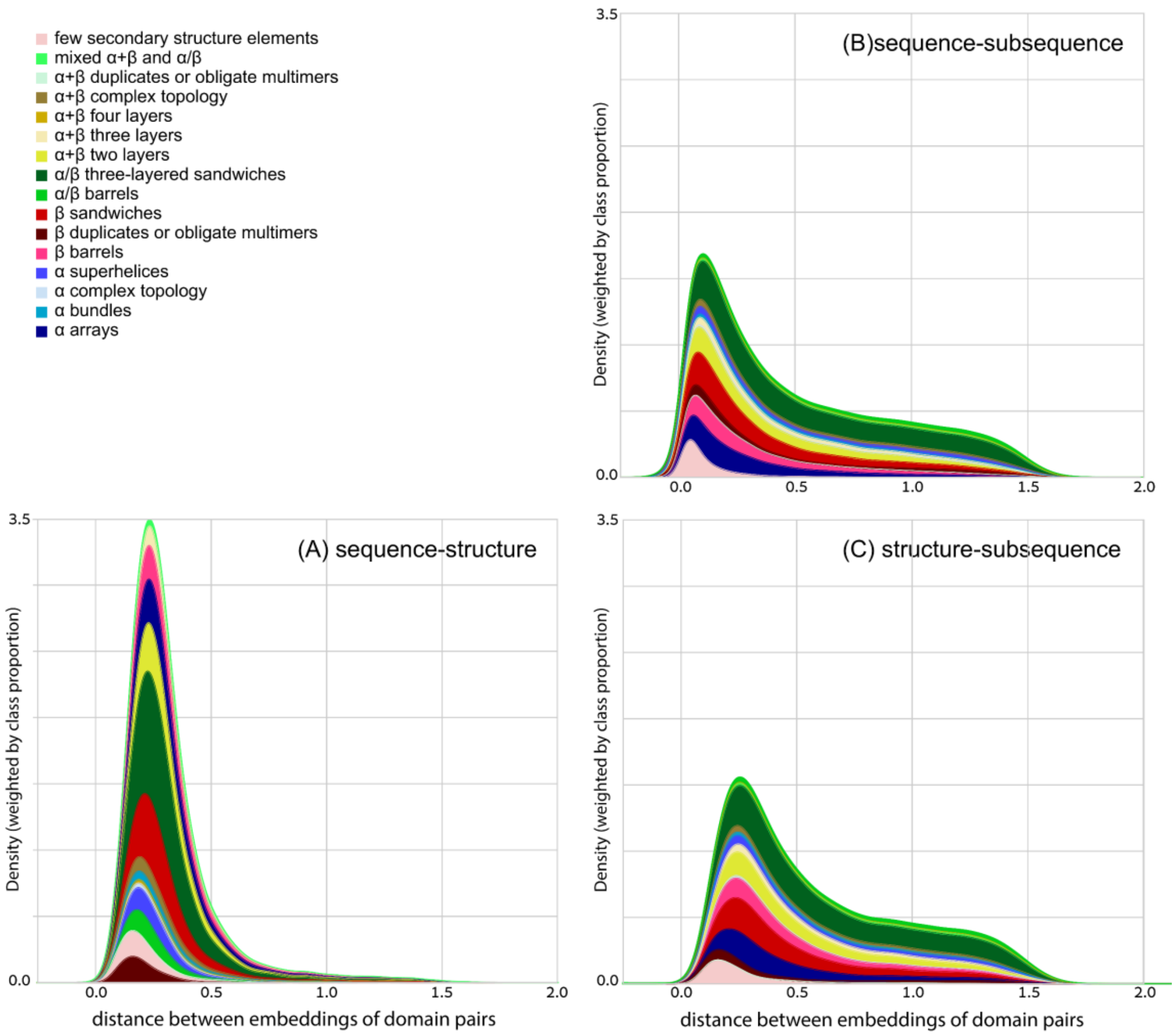

Figure S28: Distributions of the distances between CLSS-sub embeddings of different modalities for domains in Dataset 1. The distributions are grouped and colored by ECOD architecture.

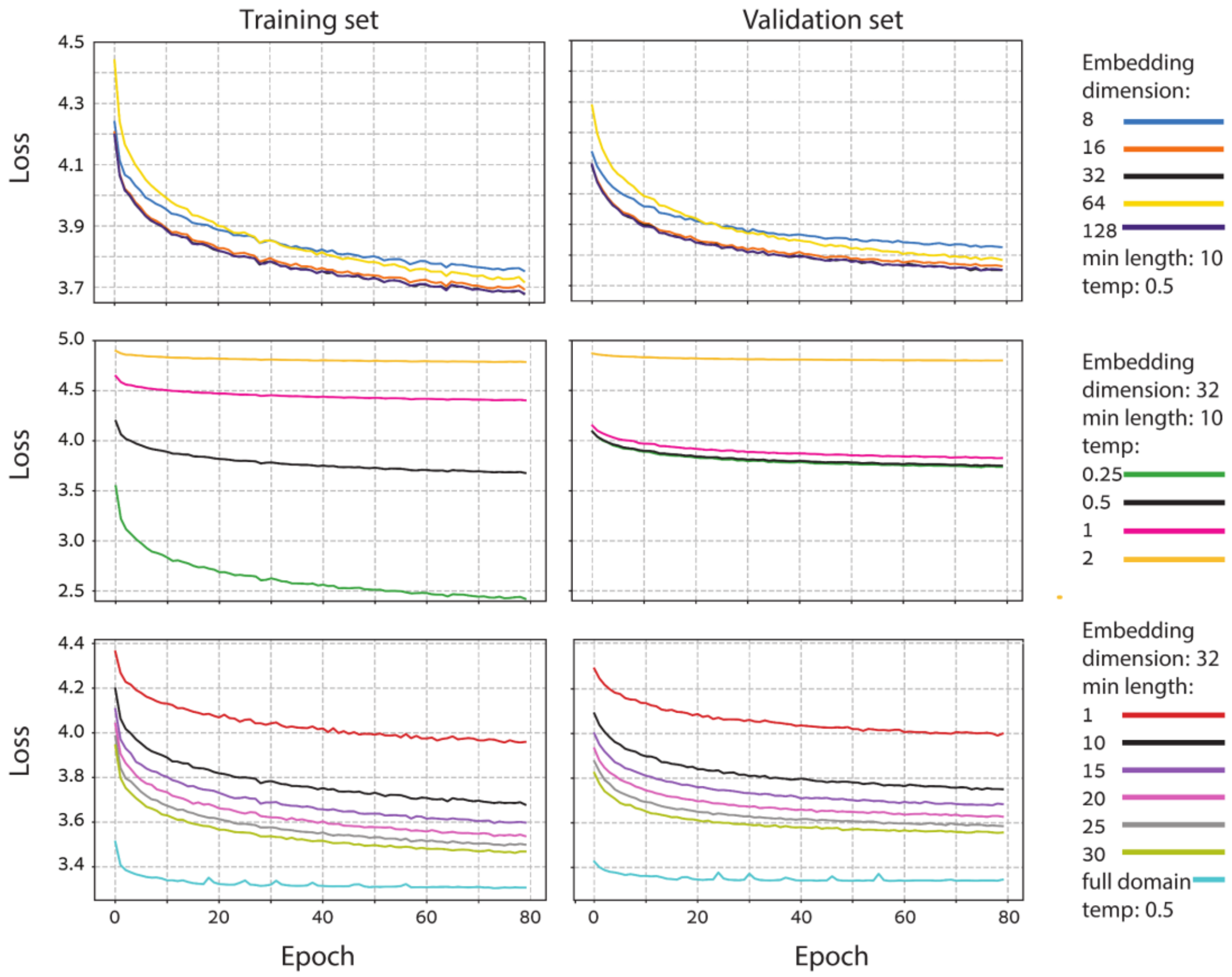

Figure S29: Training set and validation set loss graphs for different trained CLSS models during the 80 epochs of training. The rows show the parameter that we vary among the considered models: the embedding dimension, the temperature, and the min_l variable determining the lower bound on the length of the subsequence sampled.

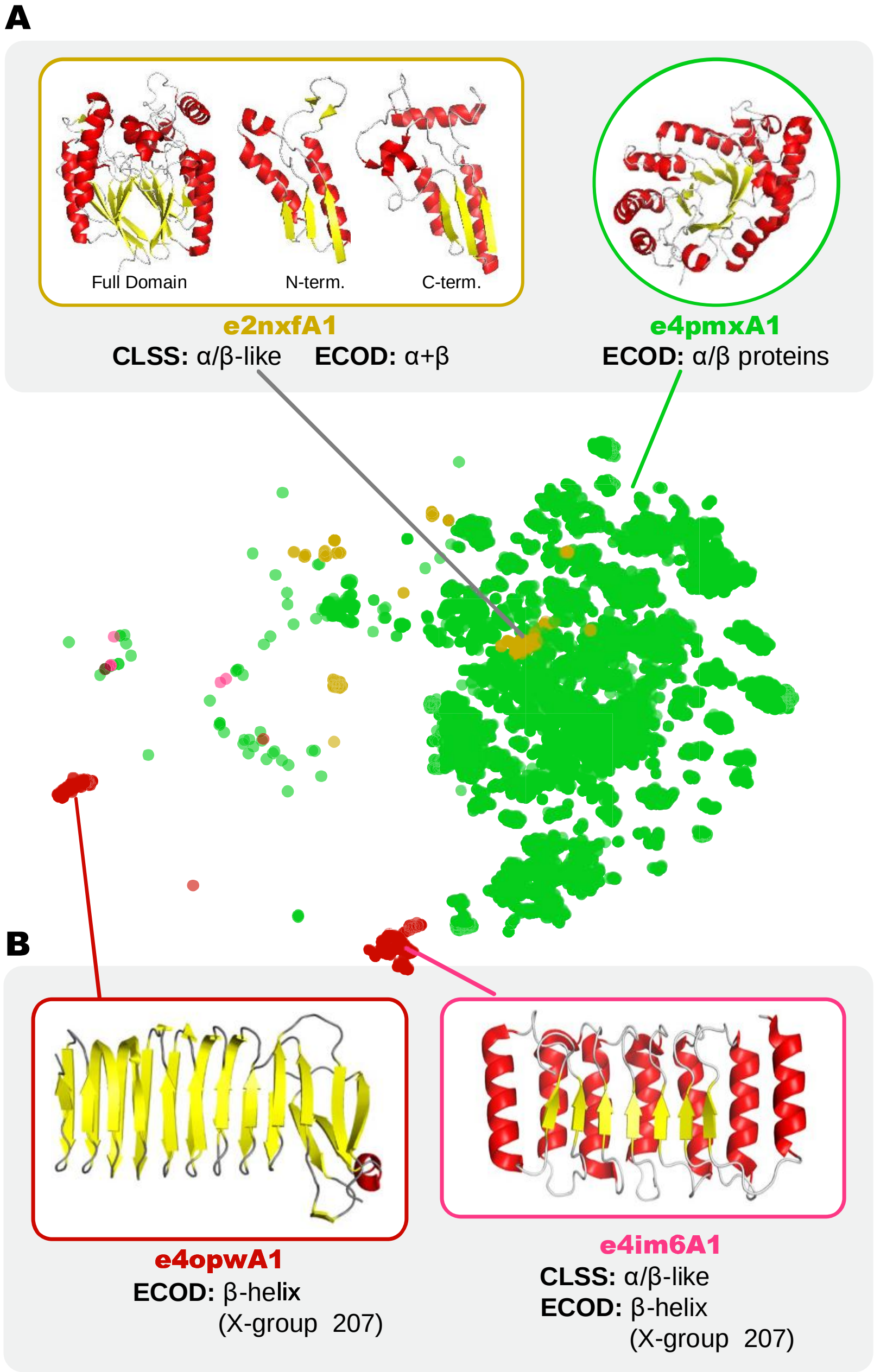

Figure S30: Example comparisons between the ECOD hierarchy and CLSS-sub maps. (A) The localization of some α+β 4-layer sandwiches (ochre) with α/β-folds (green). The CLSS-sub grouping reflects the β-α-β-α-β elements that are contained within their structures. (B) The splitting of X-group 207, which is classified by ECOD as having a β-architecture. Domains positioned near the α/β region (green) have clear signatures of α/β-structure. These examples, and another described in Figure S31, highlight the limitations of hierarchical classifications and present three candidate events of structural evolution within and between protein X-groups. See ***Supplementary Text*** *for more details.*

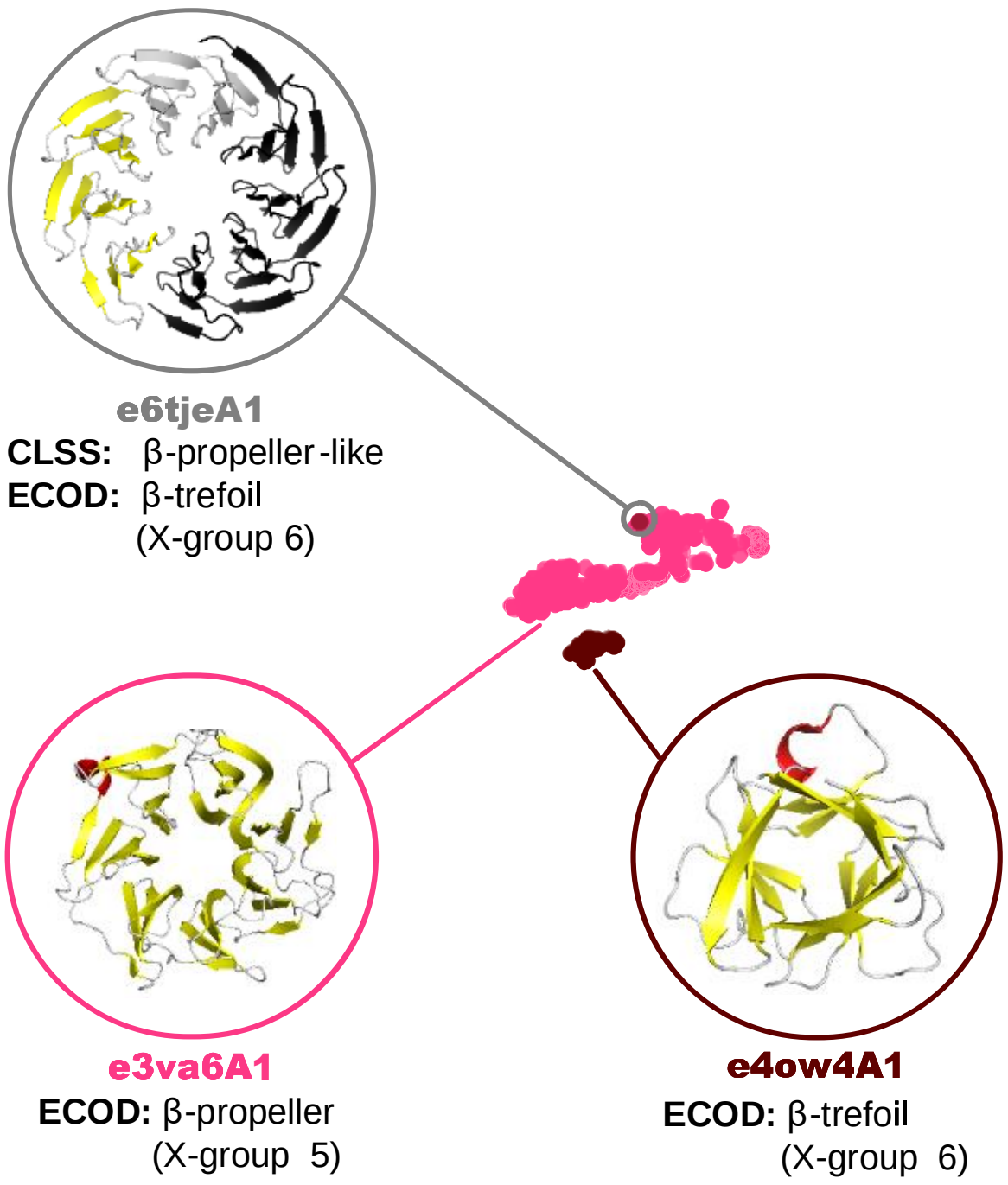

Figure S31: Another example comparison between the ECOD hierarchy and CLSS maps highlighting the challenge of automatic classification of a designed protein with sequence-based HMMs. In ECOD version 291, e6tjeA1 is part of a designed β-propeller with β-trefoil-like sequence characteristics. See ***Supplementary Text*** *for more details.*

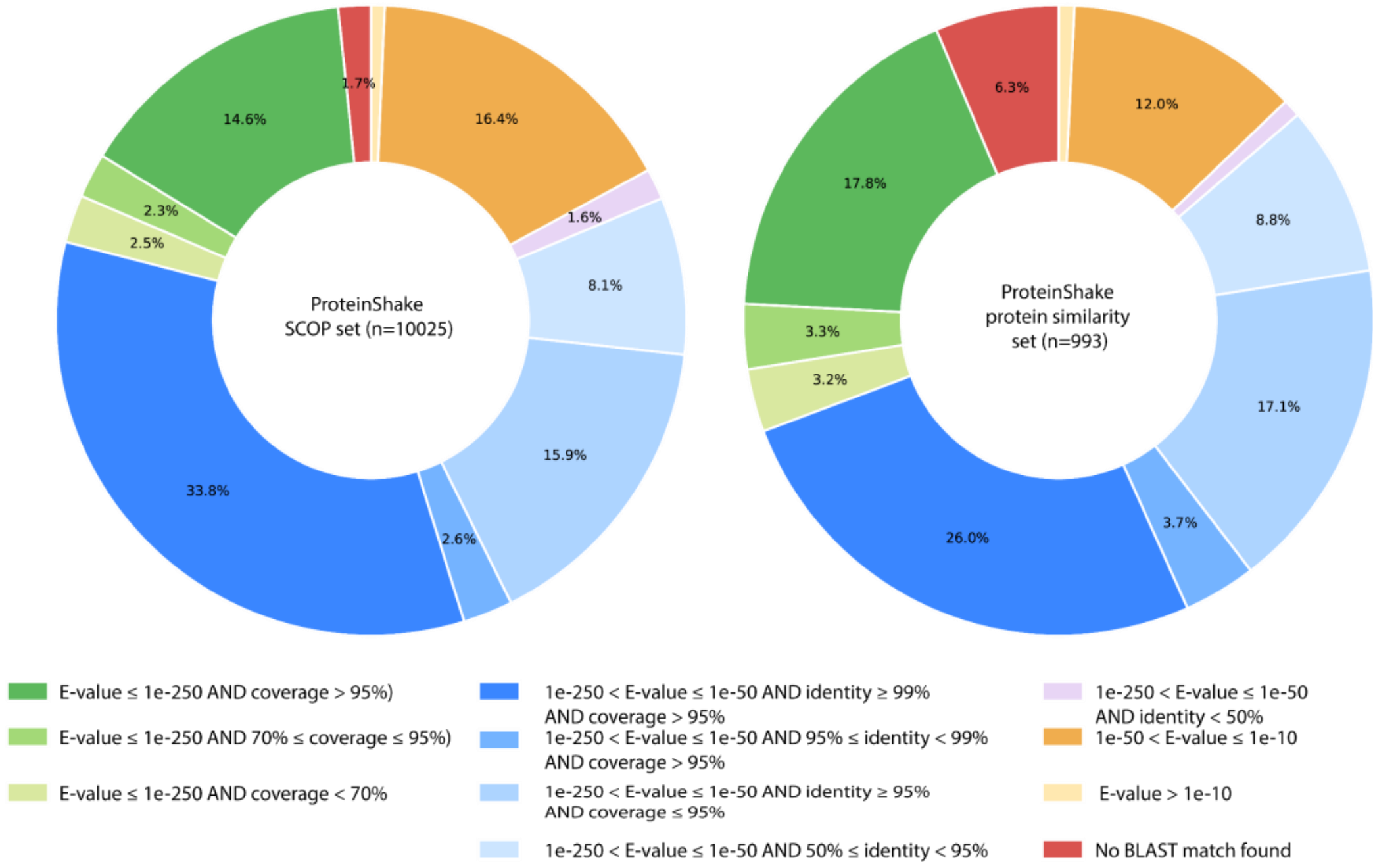

Figure S32: Identifying the ProteinShake domains (on the left, set of SCOP domains to be classified, and on the right, set of domains with structural similarities) in the training and validation sets of ECOD domains used in CLSS training. We searched using BLAST in a DB we created of the exhaustive set of ECOD domains that was used for training/validation (xxx and 50,000 respectively), and show the breakdown of the cases of found domains. As expected from the exhaustive nature of the (unlabeled) training set, most domains used in the ProteinShake datasets can be found at some level of similarity within them.

Supplementary text

Comparisons between CLSS maps and hierarchical classifications can highlight transitional proteins

Are differences between hierarchical classifications and CLSS-sub maps due to the limitations of CLSS, or do they reveal *bona fide* domain features that are not well-captured by hierarchical classification? To explore this question, **Figures S30, S31** shows instructive comparisons between ECOD and CLSS.

One example of a difference between the two approaches is the localization of α+β four-layer sandwiches within the greater α/β cluster on the CLSS-sub map (**Figure S30A**; marked in ochre). Inspection of the structure, however, affirms the CLSS clustering by revealing significant α/β-character. Domain e2nxfA1 (X-group 246), for example, has two juxtaposed β-α-β-α-β motifs that form the core of the fold. However, unlike a Rossmann domain – in which the two β-α-β-α-β motifs form a single extended β-sheet with a "doubly wound" 3-2-1-4-5-6 strand order – the β-α-β-α-β motifs of this domain pack against each other, face-to-face, to form the four-layer α-β-β-α sandwich structure [29]. The localization of the α+β four-layer sandwiches within the larger embedding space occupied by the Rossmannoid folds suggests the evolutionary potential for β-α-β-α-β motifs to interconvert between the two possible packing arrangements, albeit with a clear preference for β-strand edge-to-edge packing.

A second example of a difference between CLSS-sub embeddings and the ECOD hierarchy relates to the classification of single-stranded right-handed β-helices (X-group 207), hereafter referred to as β-helices. Although β-helices are classified as β proteins by ECOD, β-helix domains belonging to H-group 207.1 are located near α/β proteins on the CLSS map (green colored embeddings), whereas β-helix domains belonging to H-group 207.2 are located near other β-proteins, as would be expected (**Figure S30B**). Inspection of the structures once again affirms the CLSS-sub embeddings: 207.1 β-helices do, in fact, have significant α/β-character. The ECOD hierarchical classifications scheme permits only a single architecture classification per grouping, and the β-helix highlights how this limitation can be misleading for groups with diverse structures. If H-groups 207.1 and 207.2 are indeed related, as ECOD suggests, the β-helices represent a remarkable case of protein structure exploration, bridging the space of β-folds with that of α/β-folds.

In short, differences between hierarchical classification schemes, represented here by the ECOD database, and CLSS-sub seem to reflect the rigidity inherent to hierarchical classification. These examples suggest that protein space is more continuous than hierarchal in nature.

A third example (**Figure S31**) highlights the challenges facing sequence-based HMMs when used for classification. We have previously observed sequence and structure connections between the β-trefoils (X-group 6) and β-propellers (X-group 5) [1,2]. Consistent with these results, the CLSS embeddings of these two β-folds form adjacent but distinct clusters. The domain e6tjeA1 annotated by the automatic ECOD pipeline as a β-trefoil, clusters with the β-propellers in the CLSS embedding space across all three modalities (**Figure S31**). Inspection of the structure reveals that 6tje is a Rosetta-designed 9-bladed β-propeller called Cake5 [3], consistent with the CLSS-sub embedding. The classification of e6tjeA1 by the automatic ECOD pipeline (version

291) as a β-trefoil suggests that these folds are indeed evolutionarily related and provides an instructive example: Briefly, Cake5 must dimerize to form a complete β-propeller and the ECOD pipeline divided one chain of Cake5 into two separate domains: The first “β-trefoil domain” (e6tjeA1) comprises 3 β-propeller blades with a total of 12 β-strands, the same number as in a canonical β-trefoil. The second “domain” (e6tjeA2) encompasses just 7 β-strands and is correctly classified by ECOD as a β-propeller, despite being a smaller segment. As Cake 5 is a designed sequence (which can differ significantly from known protein families of the same fold), a repetitive sequence (where the number of repeats can vary between related domains, complicating the identification of domain boundaries), and a homo-oligomer (where the full domain is split across two chains), it is an exceptionally difficult protein to classify automatically.

Table S1 Legend*: Lists of ECOD domains in Dataset1 and CATH domains in Dataset2*

Table S2 Legend*: The co-factor family label that we associate with the different PDB co-factors. The table shows our groupings of the PDB co-factors along with their co-factor family name.*

References

1. Tenorio CA, Longo LM, Parker JB, Lee J, Blaber M: **Ab initio folding of a trefoil-fold motif reveals structural similarity with a β-propeller blade motif**. *Protein Science* 2020, **29**:1172-1185.
2. Longo LM, Kolodny R, McGlynn SE: **Evidence for the emergence of β-trefoils by ‘Peptide Budding’from an IgG-like β-sandwich**. *PLoS computational biology* 2022, **18**:e1009833.
3. Mylemans B, Laier I, Kamata K, Akashi S, Noguchi H, Tame JRH, Voet ARD: **Structural plasticity of a designer protein sheds light on β-propeller protein evolution**. *The FEBS Journal* 2021, **288**:530-545.
